# K_5_Ir: reduced iridium stabilized at high pressure

**DOI:** 10.1039/d6sc03528k

**Published:** 2026-09-25

**Authors:** Douglas H. Fabini, Romana-Iryna Martyniak, Angelika D. Rosa, Stella Chariton, Eric A. Riesel, Emin Mijit, Vitali B. Prakapenka, Danna E. Freedman

**Affiliations:** a Department of Chemistry, Massachusetts Institute of Technology Cambridge Massachusetts 02139 USA danna@mit.edu; b PSI Center for Neutron and Muon Sciences, Paul Scherrer Institute 5232 Villigen PSI Switzerland douglas.fabini@psi.ch; c European Synchrotron Radiation Facility 38043 Grenoble Cedex 9 France; d Center for Advanced Radiation Sources, University of Chicago Argonne Illinois 60439 USA

## Abstract

Alkali binary compounds offer a way to expand our understanding of the periodic table. Specifically, the redox inert nature of alkali cations, even within some intermetallic compounds, enables one to access exotic oxidation states. Iridium presents a test case, as it is the transition metal best positioned to form a trianion, which would be the most highly charged transition metal anion reported. Pressure, known to modulate both reactivity and electronic structure to stabilize exotic phases, is a promising path to this species. Others predicted that reduction of Ir with alkali metals at high pressure may yield phases containing the Ir^3−^ ion. We tested these predictions by reacting a K-rich mixture of K and Ir at 19.5(6) GPa and 493 K. This reaction yields K_5_Ir, which adopts the rare but simple BaSn_5_ crystal structure. Based on hybrid functional electronic structure calculations and net atomic charge analysis, K_5_Ir is predicted to be a semimetal with a carrier density ∼10^20^ cm^−3^ which features strongly reduced Ir (confirmed by Ir L_3_-edge X-ray absorption spectroscopy) and both oxidized and neutral K on different sites. The (negative) net atomic charge of Ir in K_5_Ir exceeds those of Pt^2−^ in Cs_2_Pt and Ir(iii−) in putative Na_3_[Ir(CO)_3_]. First-principles crystal structure prediction indicates that several other K–Ir compounds await discovery.

## Abstract 2

Pushing electrons uphill: Potassium and iridium react at high pressure to form K_5_Ir. Iridium in this phase extends the series of reduced 5d transition metals beyond the auride (Au^–^) and platinide (Pt^2–^) anions in semiconducting CsAu and Cs_2_Pt.

## Graphical abstract

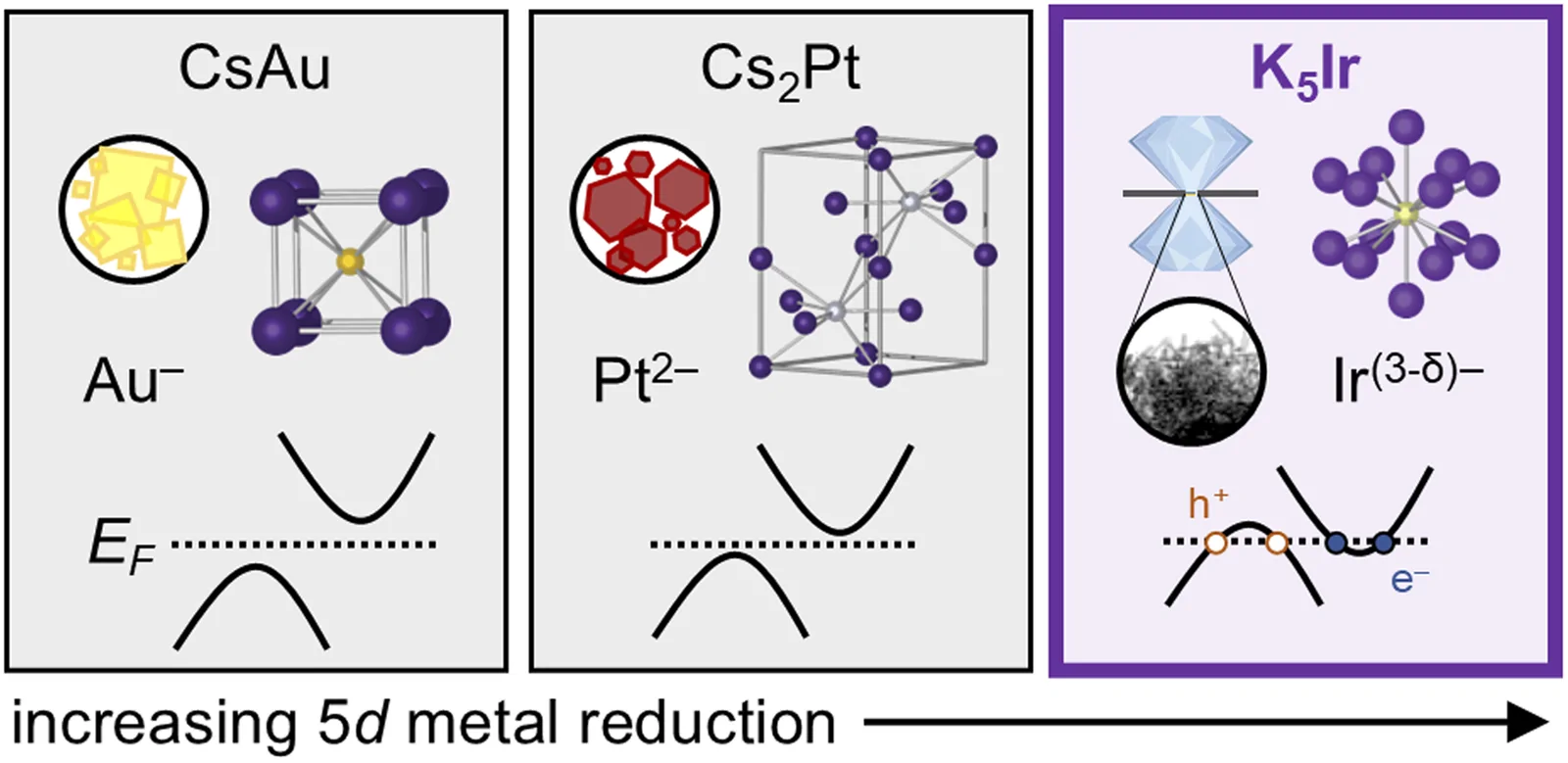

## 1 Introduction

High pressure science^1–6^ has expanded our understanding of the electronic configurations^7^ and resultant properties that are possible in simple metals,^8^ magnets,^9,10^ and the core of the earth.^11,12^ Application of pressure not only favors reduced crystal volumes but more compact electronic states, allowing sometimes for peculiar phases. Prior work demonstrated or predicted transitions from intuitive states of matter to unintuitive ones upon compression. For example, metallic sodium becomes insulating^8^ while insulating sodium chloride is predicted to metallize,^13^ liquid iron becomes diamagnetic,^14^ simple free electron-like alkali metals adopt complex structures,^15–17^ and progress has been made towards the metallization of solid hydrogen.^18–21^

Materials containing reduced iridium are an intriguing target, as a monatomic, closed-shell iridide trianion (Ir^3−^, [Xe]6s^2^4f^14^5d^10^) would be the most highly charged transition metal anion known, and the relativistically enhanced electron affinities of the late 5d metals^22,23^ may allow for such a configuration to be realized. Iridium adopts the widest range of oxidation states known,^24^ from (IX+) in gas-phase [IrO_4_]^+^^25^ to (III−) in putative Na_3_[Ir(CO)_3_], which has not been structurally characterized.^26^ As for many reduced metal complexes,^27^ the Ir(iii−) oxidation state in the purported carbonyl complex reflects the formal rules for electron counting^28,29^ and does not necessarily indicate complete valence charge separation between the metal and its ligands (*vide infra*).^30^ Unlike neighboring Pt and Au, which exhibit rich anionic chemistry and afford several colorful auride (Au^−^) and platinide (Pt^2−^) semiconductors,^31–39^ no monatomic anions of Ir have been reported. Alkalides,^40,41^ thallides,^42^ tetrelides,^43,44^ and metal clusters^45,46^ are other fascinating examples of mono- and poly-atomic metal(loid) anions in solids exhibiting full or partial charge separation.

By analogy to semiconducting CsAu^31^ and Cs_2_Pt,^38^ Pyykkö and Xu proposed reduction of iridium with cesium as a possible route to the closed-shell Ir^3−^ anion over a decade ago.^30^ However, the reactivity of alkali metals with transition metals (groups 3–11) is extremely limited, hampering our ability to prepare and learn from new transition metal anions. For example, the only such potassium-containing binary compounds reported at ambient pressure are KAu_5_, KAu_2_, K_2_Au_3_, and K_*x*_Pt (*x* < 0.5).^47–50^ Conversely, the only such iridium-containing compounds reported are LiIr and LiIr_3_.^51^ Promisingly, pressure has shown to be an effective method for eliciting reactivity between some pairs of these otherwise immiscible sets of elements, yielding ordered phases of lithium or potassium with nickel, copper, or silver^52–57^ and even slight dissolution of potassium in elemental iron.^58^

Brgoch and coworkers quantitatively studied the possibility of the monatomic Ir^3−^ anion in a solid using first principles crystal structure prediction (CSP) at high pressures.^59,60^ CSP at the fixed composition K_3_Ir revealed a semiconducting phase with a negative formation enthalpy above ∼10 GPa.^59^ CSP at several fixed compositions in the Rb–Ir and Cs–Ir systems suggested a semiconducting Rb_3_Ir phase may be stable above a similar threshold pressure while the most stable Cs_3_Ir phase is expected to be weakly metallic.^60^ To our knowledge, no realized binary compounds of iridium and alkali metals heavier than lithium, nor contacts between these elements, have been reported until now.

## 2 Results & discussion

### 2.1 Synthetic procedures & product characterization

In pursuit of the hypothetical, monatomic iridide (Ir^3−^) anion, diamond anvil cells (DACs) were loaded with K-rich mixtures of elemental K and Ir and pressurized to around 20 GPa with soft K acting as the pressure-transmitting medium. Based on the reported fixed-composition crystal structure predictions for the K–Ir, Rb–Ir, and Cs–Ir systems,^59,60^ K and Rb appeared similarly promising, and we selected the less reactive metal as an entry point. Full experimental and computational details are provided in the SI. Briefly, iridium powder was loaded into the drilled cylindrical sample space of a pre-indented rhenium gasket. Freshly cut potassium was then loaded in a nitrogen glovebox by spreading a small quantity on the culet of the top diamond and closing the DAC such that excess potassium is extruded out of the sample space *via* the contact between the pavilion of the diamond and the gasket. ***CAUTION: Potassium metal reacts violently with water and forms reactive peroxides in air. Appropriate measures must be taken for safe handling, cleaning, and disposal.*** Given this loading procedure, the precise K : Ir ratios are not known, but are estimated to be quite K-rich in all cases based on the approximate volumes of Ir loaded and the initial sample space volumes. Samples prepared in this way are air-tight at least on the timescale of many months so long as they are pressurized to ∼2 GPa before removal from the glovebox. Samples were compressed to ∼20 GPa before whole-cell or laser heating.

**Fig. 1:**
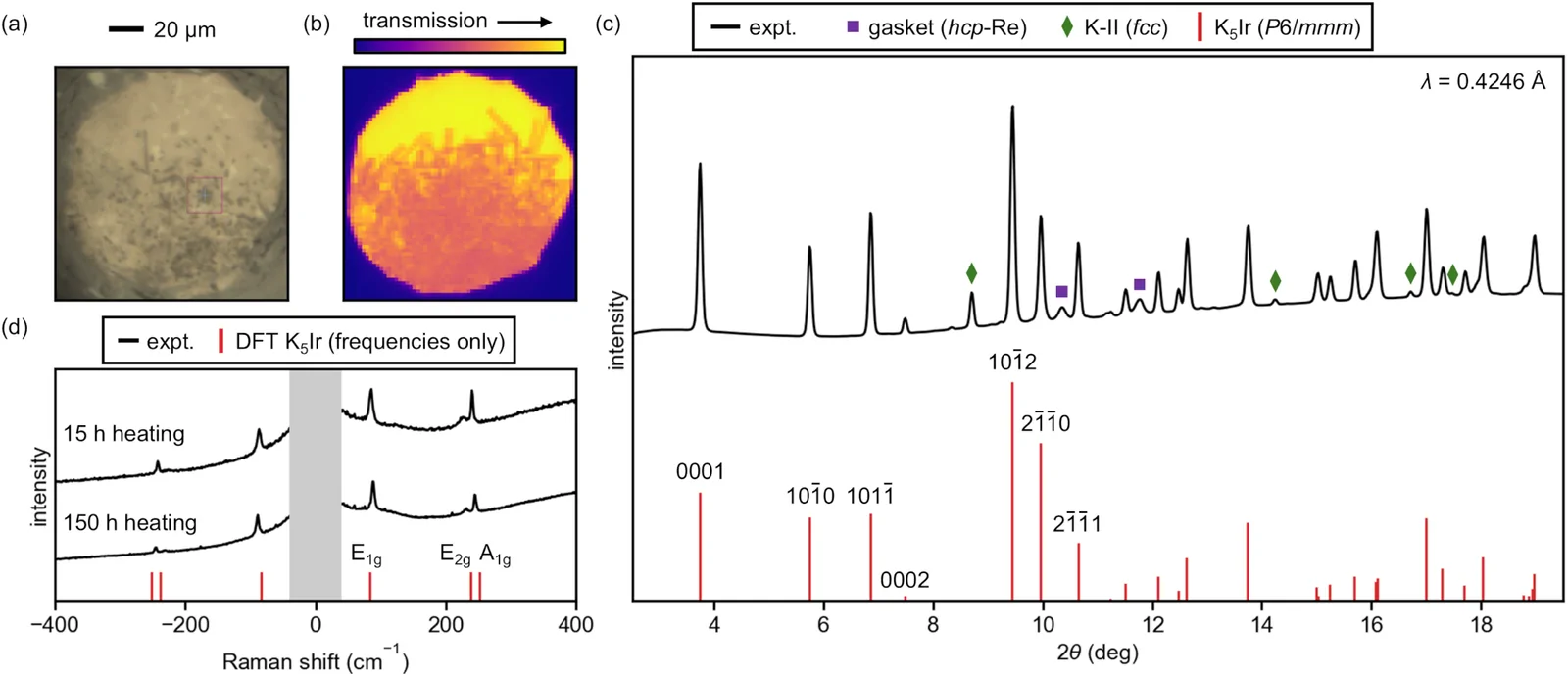
Reaction products at 19.5(6) GPa. (a) Optical microscopy shows dark-colored needles in a light-colored matrix. (b) Synchrotron X-ray imaging above the Ir L_3_-edge (ESRF BM23) shows reduced transmission through the dark needles and suggests many additional needles are hidden at visible wavelengths behind the metallic matrix phase. (c) Spatially-averaged synchrotron X-ray microdiffraction (APS 16-ID-B, see Fig. S5) is explained by a proposed K_5_Ir phase, excess K-II, and the Re gasket. Miller indices of low angle reflections for the K_5_Ir model are annotated. Bragg intensities match those from the DFT-relaxed K_5_Ir model (PBE) reasonably well, but orientation-averaging is imperfect for this collection of crystallites. Results from Rietveld refinement of the model against these data are shown in Fig. S8 and Tables S3–S5. (d) Three Raman modes are observed whose frequencies match those from DFT (PBE) for the proposed K_5_Ir model. The elastic line is masked in gray and background fluorescence of unknown origin is visible.

Samples additionally containing KBr or MgO thermal insulators and subjected to laser heating above the estimated melting point of iridium (∼3600 K at 20 GPa)^61^ displayed broad Raman scattering features after heating (Fig. S1) but negligible changes in laboratory X-ray diffraction (Table S1). Whole-cell heating under dynamic vacuum for 15 h above the reported potassium melting point (∼450 K at 20 GPa)^62^ yielded negligible changes by laboratory X-ray diffraction, but sharp signals in Raman spectra hinted at the formation of small quantities of unknown products (Fig. S1). Prolonged heating (150 h) under the same conditions yielded dark-colored needles up to 20 µm in length surrounded by a predominantly bronze-colored matrix at 19.5(6) GPa (Fig. 1a, additional sample characterization in Fig. S2–S6). The color of the matrix is consistent with the reported optical properties of elemental K at this pressure,^63,64^ and X-ray imaging and absorption spectra at the Ir L_3_-edge (BM23, European Synchrotron Radiation Facility) confirm that the dark needles contain iridium (Fig. 1b) and the matrix does not (Fig. S6).

Synchrotron X-ray microdiffraction (16-ID-B, Advanced Photon Source) is shown in Fig. 1c. Absence of Bragg reflections from Ir (fcc) and presence of those from K-II (fcc) suggest a reaction in which Ir is limiting. After accounting for the gasket and excess K, the remaining reflections could be clearly indexed to a hexagonal cell with no systematic absences. This allowed for the 16 trigonal and hexagonal space groups with no centering, glide planes, or screw axes. Several structures of known binary compounds were collected from the Inorganic Crystal Structure Database^65^ based on compatibility with this set of space groups and similarity of the *c*/*a* ratio. These structures were populated with unique permutations of K and Ir and optimized with density functional theory (DFT-PBE). The resulting Bragg reflection intensities and positions were compared with those from experiment, and the formation enthalpies were compared across the candidates. K_5_Ir in the BaSn_5_ structure type could be quickly identified as an excellent match to the data.

Zone-center phonons and their irreducible representations were calculated for this candidate phase, revealing excellent agreement between DFT-computed Raman frequencies and those observed experimentally (Fig. 1d). Further, evolutionary crystal structure prediction based on DFT indicates that this candidate phase lies on the zero temperature convex hull of the K–Ir system at 20 GPa (*vide infra*). Lastly, computed dynamic and elastic stability do not exclude this model (Fig. S7 and Table S2). Based on this agreement of diffraction (both Bragg angles and intensities), Raman frequencies, and DFT-computed thermodynamics, we conclude K_5_Ir in the BaSn_5_ structure is the product.

The results of Rietveld refinement of the crystal structure model against X-ray diffraction are presented in Fig. S8 and Tables S3–S5 and are available from the Cambridge Crystallographic Data Centre (deposition code 2521670). Several features of this high pressure dataset pose challenges relative to typical ambient pressure powder diffraction. This ensemble of K_5_Ir needles has too many crystallites for multi-grain crystallographic analysis but too few for complete powder averaging of orientations. The background is strong and structured. Lastly, the peak profile is challenging to fit well over the entire observed *Q*-range. Due to these limitations, atomic displacement parameters should be interpreted with care. Nevertheless, the only free atom position parameter for this structure, *z* for one of the K sites, is robust to refinement strategy and to the inclusion or omission of absorption corrections, and it agrees with the value from DFT-PBE optimization reasonably well (Table S5). Our final crystallographic model fits the data with weighted profile R-factor, *R*_wp_ = 2.325%, and expected *R* factor, *R*_exp_ = 1.529%, corresponding to a goodness-of-fit *R*_wp_/*R*_exp_ = 1.53. Detectable partial substitution of Ir on either K site or of K on the Ir site could be excluded by Rietveld refinements with the sum of the occupancies of the site in question constrained to one.

### 2.2 Crystal structure of K_5_Ir

**Fig. 2:**
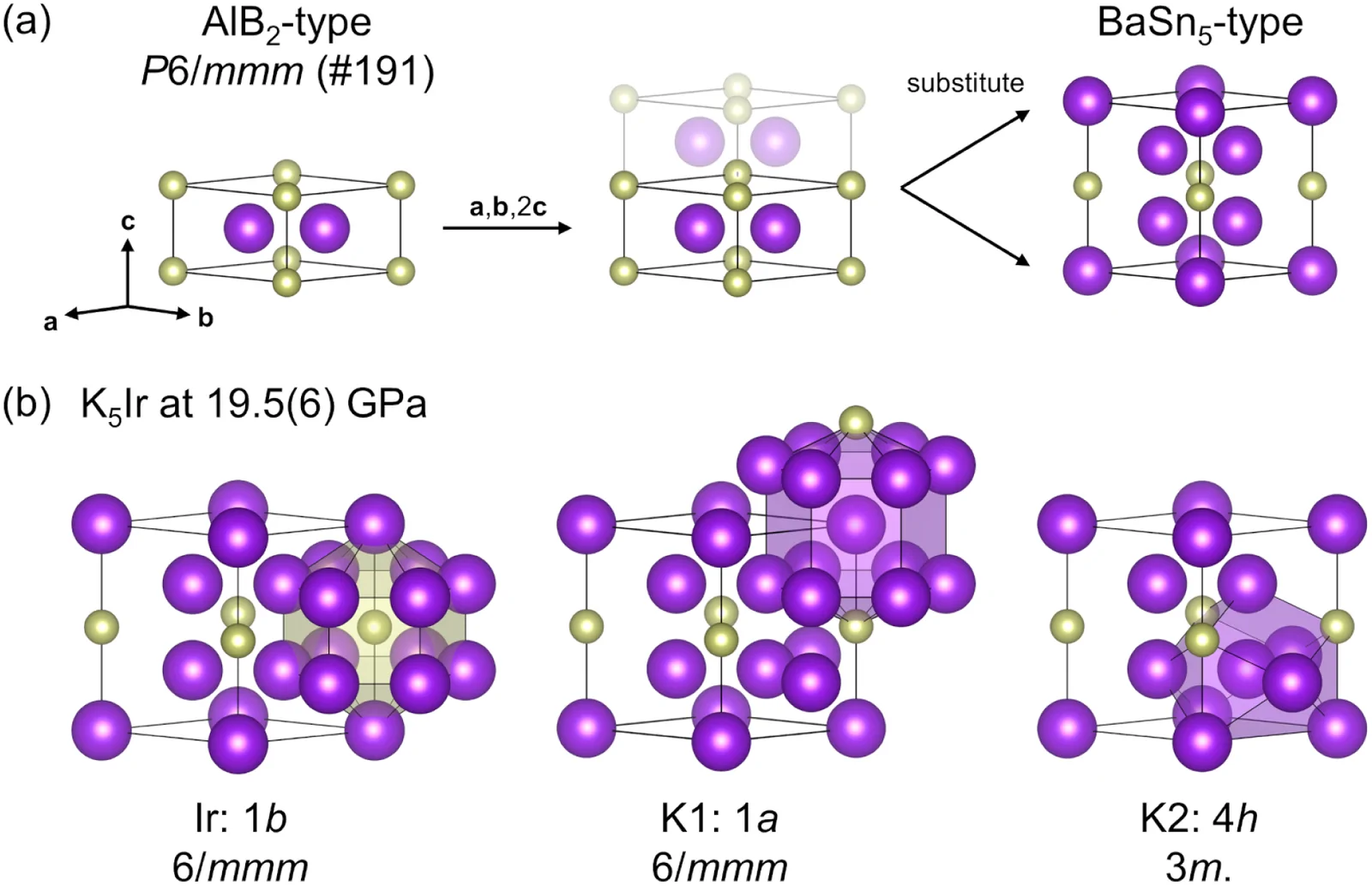
Crystal structure of K_5_Ir at 19.5(6) GPa. (a) Relationship between the AlB_2_ aristotype and the BaSn_5_ hettotype adopted by K_5_Ir. (b) Crystal structure model of K_5_Ir, with the coordination polyhedra around each site depicted. Potassium atoms are purple and iridium atoms are golden. Wyckoff labels and site symmetries are annotated.

The structure type adopted by K_5_Ir at 19.5(6) GPa (space group *P*6/*mmm*, Pearson symbol *hP*6) has been observed once previously for superconducting BaSn_5_ (ref. 66) and is a substitution derivative of the ubiquitous AlB_2_-type (Fig. 2a).^66,67^ The structure and the coordination environments of each unique site at 19.5(6) GPa are shown in Fig. 2b.

The unique K (1a Wyckoff site) and the Ir(1b) display bicapped hexagonal prismatic coordination. It appears that contacts between Ir and alkali metals heavier than Li have not been previously reported. Such contacts in K_5_Ir at 19.5(6) GPa are 313 pm and 325 pm to the K(4h) and K(1a) sites, respectively. The hexagonal prism around K(1a) is substantially elongated along c relative to that around Ir(1b). From the K(4h) site, contacts to other K(4h) are much shorter (282 pm within hexagonal nets, 272 pm joining eclipsed hexagonal net bilayers across the Ir coordination prism) than those to Ir(1b) or to K(1a).

### 2.3 Electronic structure and charge distribution of K_5_Ir

**Fig. 3:**
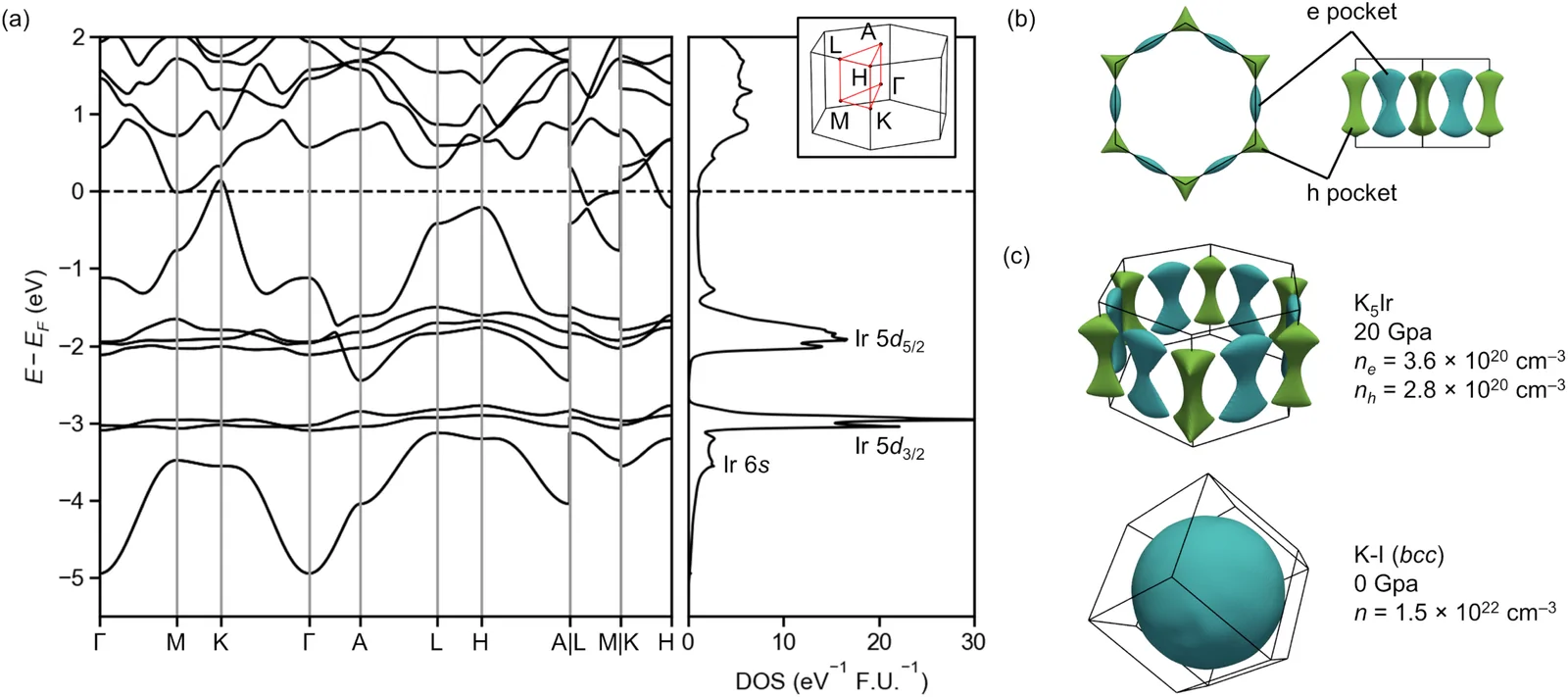
Screened hybrid functional electronic structure of K_5_Ir at 20 GPa, including the spin–orbit interaction (HSE + SOC). (a) Electronic band structure and density of states near the Fermi energy, *E*_F_. The Brillouin zone high symmetry point labels and path are inset. (b) Fermi surface, revealing small and somewhat compensated electron and hole pockets on the zone boundary. (c) Comparison of the Fermi surfaces of K_5_Ir at 20 GPa and elemental K at ambient pressure. The carrier density of K_5_Ir is predicted to be roughly 4% of that of K–I (bcc).

While semilocal DFT is often sufficient for describing crystal structures, phonons, elastic properties, and total energies of materials without strong correlations, screened hybrid functionals incorporating exact Hartree–Fock exchange can significantly improve the description of electronic structure and related derived metrics.^68^ Except where explicitly noted, we employed the HSE hybrid functional for electronic structure and the PBE semilocal functional for crystal structures, phonons, elastic properties, and total energies. The relativistic electronic band structure and density of states of K_5_Ir computed with a screened hybrid functional and spin–orbit coupling (HSE + SOC) are given in Fig. 3a. The band derived primarily from the Ir 6s orbital (Fig. S9) lies below the flat bands primarily composed of Ir 5d_5/2_ and 5d_3/2_ orbitals, suggesting Ir is reduced with respect to the elemental metal.^45^ The next two higher bands appear to cross the Fermi energy (*E*_F_) in distinct, small regions of *k*-space. Indeed, the Fermi surface (Fig. 3b) exhibits small electron and hole pockets on different portions of the Brillouin zone boundary which resemble pinched bowties with 2-fold and 3-fold symmetry, respectively. Both carrier pockets nearly connect along c* to form open wiggly tubes, reflecting somewhat 2-dimensional electronic character. By Luttinger's theorem, the electron and hole densities in K_5_Ir are both predicted to be of order 10^20^ cm^−3^ (Fig. 3c).

**Fig. 4:**
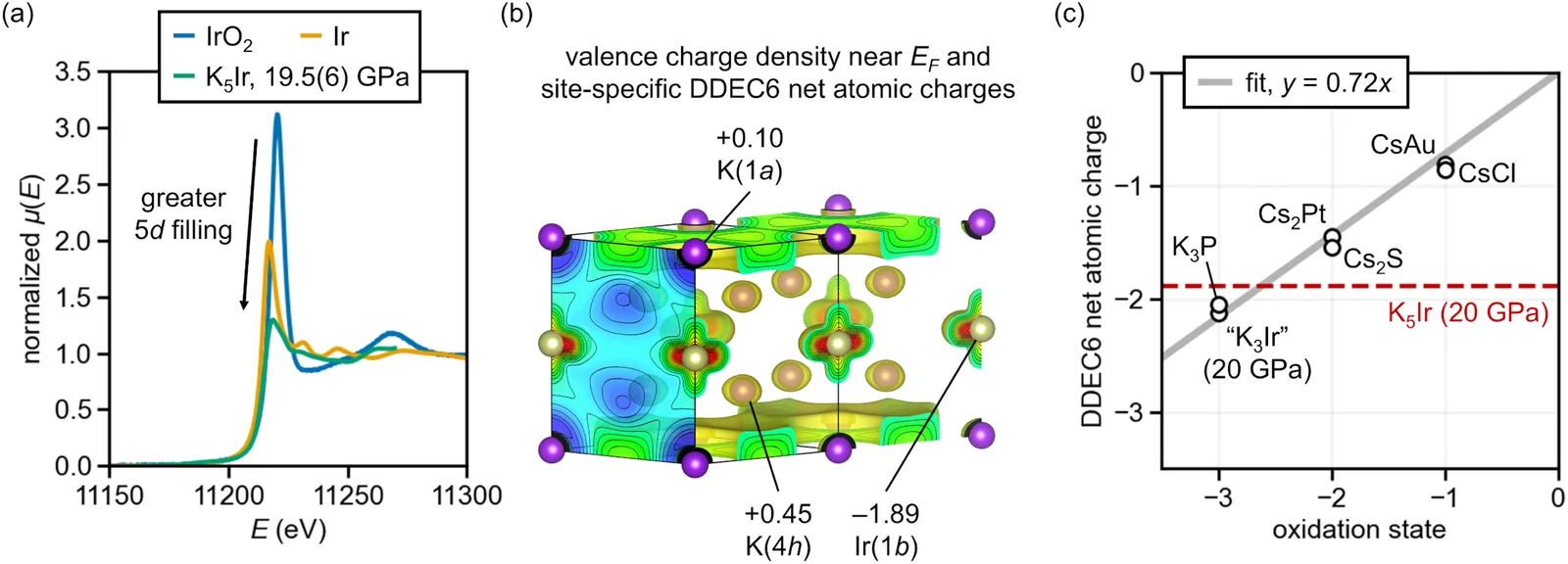
Charge distribution in K_5_Ir. (a) Iridium L_3_-edge XANES (ESRF BM23) showing a dramatic reduction in the intensity of the dipole-allowed 2p_3/2_ → 5d transition from IrO_2_ to Ir to K_5_Ir, reflecting increased filling of the 5d orbitals (reduction). (b) Computed valence charge density (DFT-PBE, see SI note) within *E*_F_ ± 100 meV and site-specific DDEC6 net atomic charges (HSE) at 20 GPa. Consistent with the nearly tubular Fermi pockets along c*, the charge density near *E*_F_ is dominated by layers along the (0001) planes encompassing the neutral K site which are bridged only weakly by Ir between the layers. (c) DDEC6 net atomic charges (HSE) and oxidation states of the anions in alkali metal-5d compounds and analogous alkali metal-3p compounds. A fit to these six phases (including the predicted K_3_Ir,^59^ indicated by quotation marks) through the origin yields the line depicted in gray. The computed net atomic charge for Ir in K_5_Ir falls between those of the 2- and 3- anions in these insulators.

K_5_Ir appears to be the alkali-richest alkali metal-transition metal binary compound reported, excluding a putative dilute alloy of Ag in Li.^69^ Given the electropositivity of K, the high electron affinities of the late 5d metals, the high K/Ir ratio, and the predicted semimetallic electronic structure, the distribution of charge in K_5_Ir is of particular interest. Measurements of iridium L_3_-edge X-ray absorption near-edge structure (XANES, BM23, European Synchrotron Radiation Facility) indicate an increased filling of the Ir 5d orbitals going from Ir(iv) in IrO_2_ (nominally [Xe]4f^14^5d^5^6s^0^) to Ir(0) in the elemental metal (nominally [Xe]4f^14^5d^7^6 s^2^) to reduced Ir in K_5_Ir, as evidenced by suppression of the dipole-allowed 2p_3/2_ → 5d transition across the series (Fig. 4a). This mirrors the trend reported for the Au L_3_-edge in Au(iii), Au(i), Au(0), and Au(i−) compounds^70^ except that complete filling of the Ir 5d orbitals is not achieved in K_5_Ir. The absorption edge corresponding to ionization from 2p_3/2_ to the continuum, which typically shifts to lower energies with reduction, is obscured by the prominent 2p_3/2_ → 5d absorption. The energy of the 2p_3/2_ → 5d transition is not a reliable diagnostic of oxidation states for compounds with dissimilar ligands and coordination environments, as it depends on the ligand field.^71^ Common metrics for the onset of this transition like the energy at half maximum or the energy at maximum slope are additionally sensitive to the core-hole lifetime. Attempts to quantify the number of 5d holes and the onset energy of ionization to the continuum by fitting to the sum of a Lorentzian (2p_3/2_ → 5d) and an arctangent step (2p_3/2_ → continuum)^70^ were unsuccessful: The Lorentzian intensity and step energy are strongly correlated when the Lorentzian becomes weak as in K_5_Ir, and they are additionally somewhat sensitive to the normalization procedure employed. Diamond artifacts in the high-pressure K_5_Ir spectrum not far above the L_3_-edge preclude fitting a more sophisticated model which includes multiple scattering of photoelectrons. The qualitative conclusion that Ir in K_5_Ir is strongly reduced with respect to Ir(0) is, however, robust to the data normalization procedure. Ir and IrO_2_ were measured at ambient pressure while K_5_Ir was measured at 19.5(6) GPa. The X-ray absorption spectrum of Ir is reported to be minimally affected by pressure^72^ and our own calculations of net atomic charges (*vide infra* and Table S6) indirectly indicate negligible pressure dependence of the electronic structures of both Ir and IrO_2_ between zero and 20 GPa, presumably reflecting their low bulk compressibilities.

As another perspective on the charge distribution, the density derived electrostatic and chemical (DDEC6) net atomic charges of each species were computed with a screened hybrid functional (HSE). While there is no physically unambiguous way to partition charge- or spin-density among atoms in molecules or solids, the DDEC6 method is reported to exhibit several favorable features including basis set independence and unique solutions.^73,74^ By this metric, the Ir site is anionic, with a net atomic charge of −1.89, while K(4h) is somewhat cationic (+0.45) and K(1a) is approximately neutral (+0.10). The distinct net atomic charges of the two K sites are reminiscent of the charge separation and distinct bonding modes observed in elemental Na and K at yet higher pressures.^8,75^

The valence charge density due to states close to *E*_F_ (Fig. 4b, DFT-PBE, see SI note) is dominated by the connected interstitial space in (0001) layers encompassing the neutral K(1a) site. These layers are weakly bridged to one another *via* the Ir, while there is little density from the cationic K(4h) towards either the (0001) layers of interstitial density or towards the Ir site (Fig. S10). The electron localization function (ELF, Fig. S11) also bears signatures of covalency in the interstitials of the (0001) layers containing K(1a). Both the charge density and the ELF suggest parallels to the situations in metallic BaPt, Ba_2_Pt, and Sr_2_Ni_3_, wherein excess electrons are delocalized in planes containing the alkaline earth.^76–78^ In a similar manner, K_5_Ir can be approximately described as “K(K^+^)_4_(Ir^3−^)·e^−^”, with the extra electron filling the otherwise-half-filled first band above the Ir 5d manifold.

**Fig. 5:**
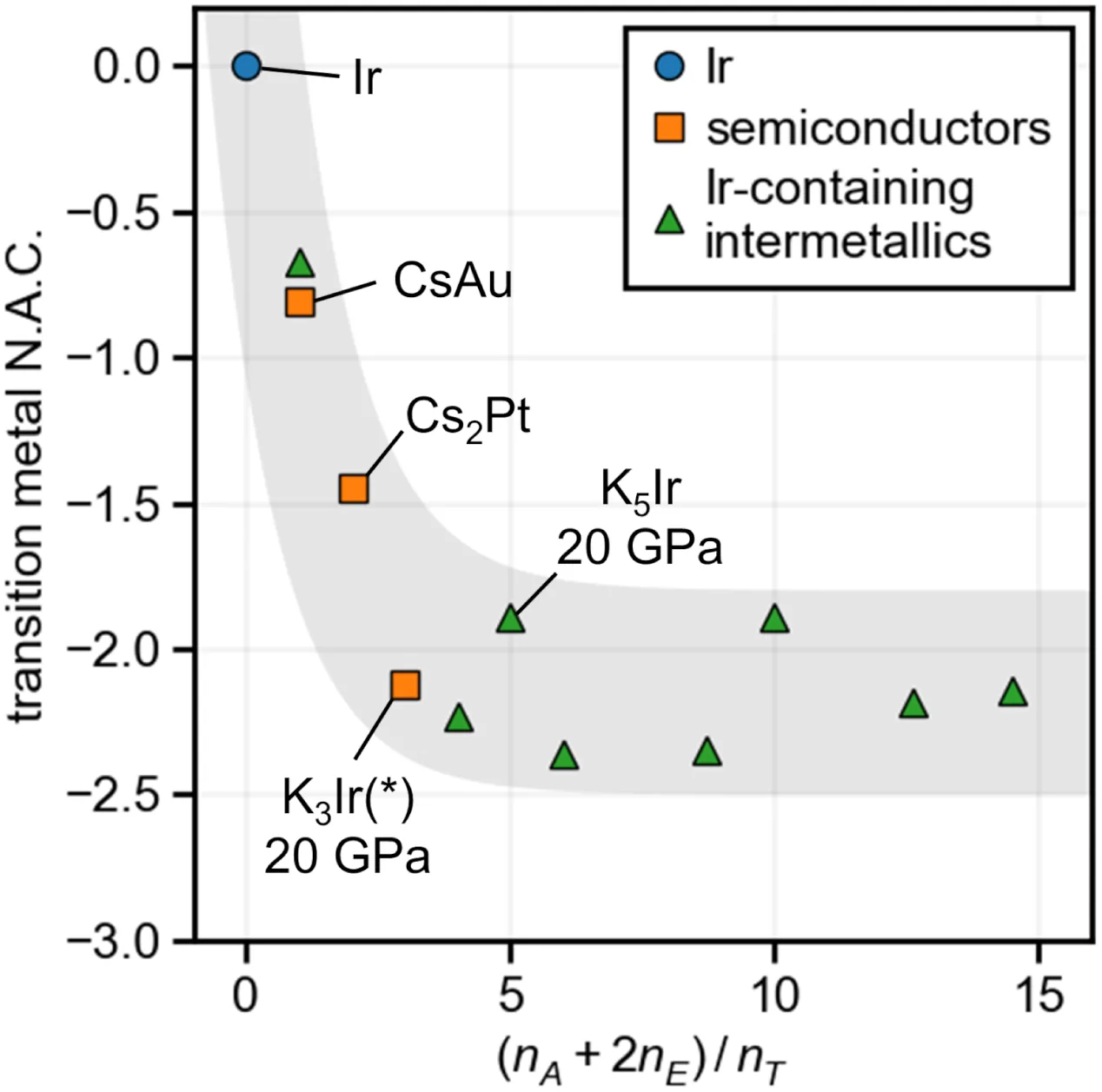
Relationship between the transition metal DDEC6 net atomic charges (“N.A.C.”) at the HSE level and the composition (*n*_A_ = number of alkali metal atoms, *n*_E_ = number of alkaline earth metal atoms, *n*_T_ = number of transition metal atoms) for the alkali- and alkaline-earth-iridium intermetallic phases in Table S11 plus elemental Ir and semiconducting CsAu, Cs_2_Pt, and hypothetical K_3_Ir. The Ir N.A.C. for the intermetallic phases is seen to saturate for Ir-poor compositions within ±12% of the value for hypothetical, charge-separated K_3_Ir.

To interpret the degree of anionic character of Ir in K_5_Ir, we computed the DDEC6 net atomic charges (HSE) for the anions in CsAu, Cs_2_Pt, hypothetical K_3_Ir,^59^ and their 3p analogs, CsCl, Cs_2_S, and K_3_P (Fig. 4c and Table S7). Tests indicate that this analysis is not significantly influenced by the choice of DDEC6 *versus* other common net atomic charges (Table S8) or by comparing to the reference phases at zero pressure *versus* 20 GPa (Tables S6 and S9). While the Ir net atomic charge (−1.89) in K_5_Ir falls short of that of Ir^3−^ in hypothetical, semiconducting K_3_Ir (−2.12), it exceeds that of Pt^2−^ in semiconducting Cs_2_Pt (−1.45) and vastly exceeds that of Ir(iii−) in putative Na_3_[Ir(CO)_3_] (−1.16), the only system reported to contain Ir(iii−) to date (Table S10).^26^ This result is consistent with the Ir L_3_-edge XANES above, which indicates nearly complete filling of the 5d orbitals. We then applied the same net atomic charge analysis to reported alkali- and alkaline-earth-iridium intermetallics, which leads to the prediction that several such alkali- or alkaline-earth-rich phases contain similarly- or even slightly more-reduced iridium, a feature which has gone unreported until now (Fig. 5 and Table S11). Across these phases, we find a straightforward relationship between composition and transition metal net atomic charge. The (negative) net atomic charge of the transition metal initially scales with the number of electrons available for donation from the s-elements in the limit of oxidation to the group valence before saturating around the net atomic charge for the iridide (Ir^3−^) anion in hypothetical K_3_Ir.

### 2.4 Thermodynamics of the K–Ir system

**Fig. 6:**
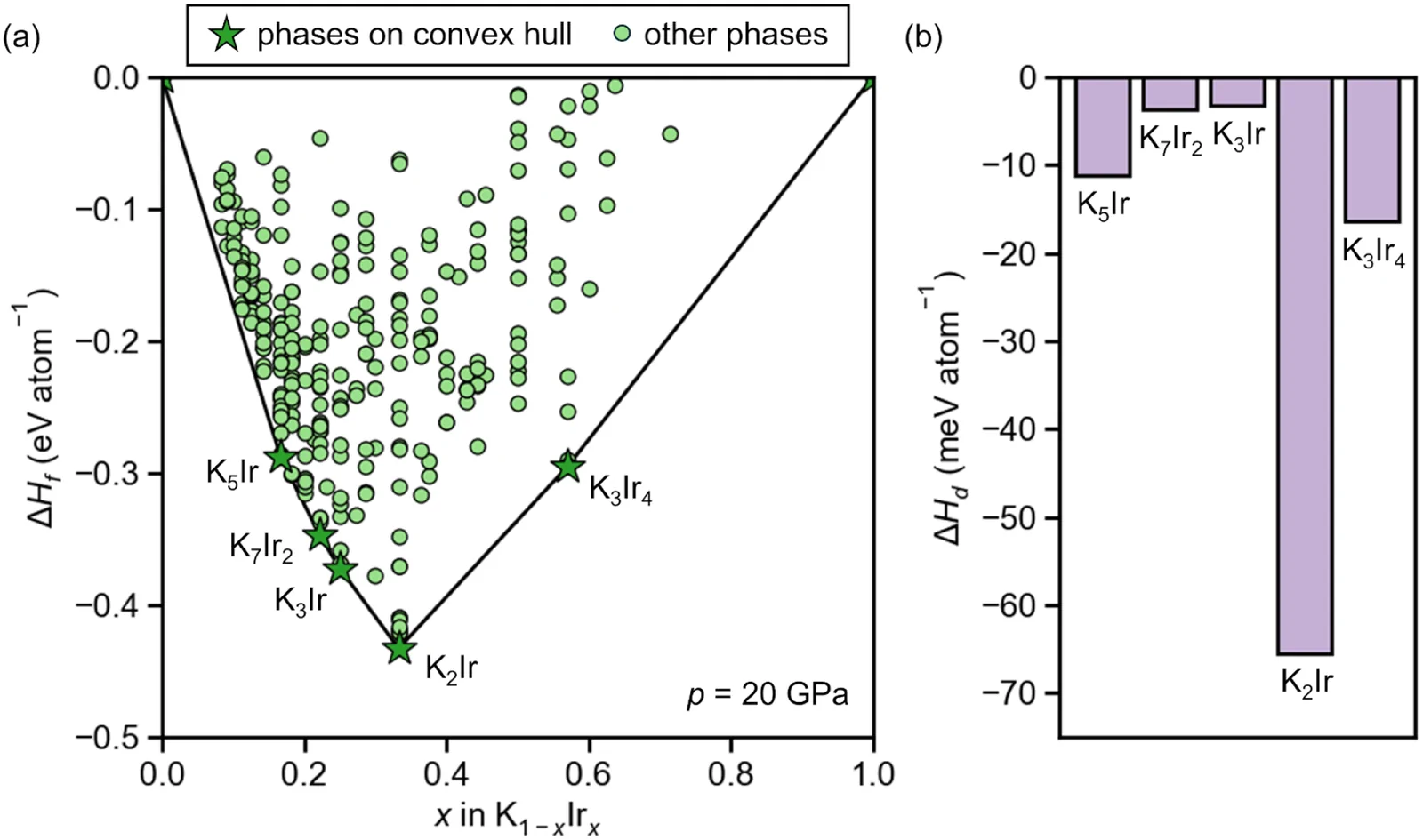
Zero-temperature crystal structure prediction (DFT-PBE) for the K–Ir system at 20 GPa. (a) Formation enthalpies, Δ*H*_f_, of candidate phases suggest several compounds beyond the K_5_Ir reported here. (b) Decomposition enthalpies, Δ*H*_d_, of the phases on the convex hull, excluding other polymorphs at the same compositions. The prediction of K_2_Ir formation at this pressure is robust, while the decomposition enthalpy of the predicted K_3_Ir semiconductor^59^ is on par with the typical effects of finite temperature and other possible sources of error.

*Post hoc* crystal structure prediction (DFT-PBE) using an evolutionary algorithm (Fig. 6a) provides further support for the proposed model of K_5_Ir, which lies on the zero-temperature convex hull at 20 GPa. Further, a two-phase mixture of K_5_Ir and K is calculated to be stable for K : Ir ratios in excess of 5, in agreement with a rough estimate of the precursor stoichiometry based on the lateral coverage and thickness of the iridium flake loaded and the initial volume of the sample space (K : Ir ratio ∼ 10). These calculations also predict that several Ir-richer K–Ir binary compounds await discovery at the same pressure. To quantify the robustness of these predictions, the decomposition enthalpy of the phases on the convex hull (excluding other polymorphs at the same composition) is computed in Fig. 6b. Of the five predicted phases, the K_3_Ir phase (which matches that initially predicted in the fixed-composition search by Brgoch and Hermus)^59^ appears to compete closely with K_7_Ir_2_ and K_2_Ir. It remains to be seen whether a charge-separated compound containing the elusive monatomic Ir^3−^ anion can be prepared at this or a different pressure or by reaction with a different alkali metal.

Lastly, we investigated the ambient pressure reactivity of K and Ir and the thermodynamics of K_5_Ir decompression computationally (DFT-PBE). In line with the lack of reports of ambient pressure K–Ir binary compounds, crystal structure prediction at zero pressure did not reveal any stable crystalline phases (Fig. S12). The formation enthalpy of K_5_Ir is predicted to be positive below a threshold pressure ∼8 GPa (Fig. S13), implying decomposition to the elements or undiscovered competing phases limited only by the kinetics of bond breakage and solid state diffusion.

## 3 Conclusions

We described the preparation, crystal structure, and electronic structure of K_5_Ir, which we synthesized by a high pressure alkali metal self-flux reaction. Ir in this phase is strongly reduced, with a nearly filled d-shell indicated by X-ray absorption spectroscopy and hybrid functional electronic structure methods. K is predicted to be somewhat oxidized on one site and approximately neutral on the other. We also reported predictions of several other thermodynamically stable compounds in the K–Ir system which may be accessible *via* similar methods and which might contain intriguing reduced iridium species.

## Author contributions

DHF: conceptualization, investigation, supervision, writing – original draft, writing – review & editing. RIM: investigation, writing – review & editing. ADR: investigation, writing – review & editing. SC: investigation, writing – review & editing. EAR: investigation, writing – review & editing. EM: investigation, writing – review & editing. VBP: investigation. DEF: funding acquisition, investigation, supervision, writing – review & editing.

## Conflicts of interest

There are no conflicts to declare.

## Supplementary Material

SC-OLF-D6SC03528K-s001

## Data Availability

CCDC 2521670 (K_5_Ir) contains the supplementary crystallographic data for this paper.^131^ Ref. 79–130 are cited in the supplementary information (SI). Other data supporting this article have been included as part of the SI. Supplementary information: experimental and computational details, additional figures and tables. See DOI: https://doi.org/10.1039/d6sc03528k.
