# Supplementary material for: K5Ir: reduced iridium stabilized at high pressure
Source: Chem Sci. 2026 Sep 25. Online ahead of print. doi: 10.1039/d6sc03528k (PMC13613704; doi:10.1039/d6sc03528k)
Supplement: Supplementary file 1 [file SC-OLF-D6SC03528K-s001.pdf]

Supplementary Information (SI) for Chemical Science.
This journal is © The Royal Society of Chemistry

Supporting Information for:

K_5Ir: Reduced iridium stabilized at high pressure

Douglas H. Fabini,[1,2*] Romana-Iryna Martyniak,[1] Angelika D. Rosa,[3] Stella Chariton,[4] Eric A. Riesel,[1] Emin Mijit,[3] Vitali B. Prakapenka,[4] Danna E. Freedman[1*]

1. Department of Chemistry, Massachusetts Institute of Technology, Cambridge, Massachusetts 02139, United States
2. PSI Center for Neutron and Muon Sciences, Paul Scherrer Institute, 5232 Villigen PSI, Switzerland
3. European Synchrotron Radiation Facility, 38043 Grenoble Cedex 9, France
4. Center for Advanced Radiation Sources, University of Chicago, Argonne, Illinois 60439, United States

* Corresponding authors. Email: douglas.fabini@psi.ch (D.H.F.), danna@mit.edu (D.E.F.)

Table of Contents

**Experimental Details**	**S3**
**Computational Details**	**S5**
**Figure S1** | Comparison of Raman spectra of products across heating methods	**S7**
**Table S1** | Sensitivity analysis for first-principles thermodynamic calculations	**S8**
**Figure S2** | Optical microscopy at key steps of sample processing	**S9**
**Figure S3** | Laboratory X-ray diffraction during compression of precursor mixture	**S10**
**Figure S4** | Laboratory X-ray diffraction at key steps of sample processing	**S11**
**Figure S5** | Spatial distribution of reaction products: X-ray microdiffraction	**S12**
**Figure S6** | Spatial distribution of reaction products: Ir L_3-edge XAS	**S13**
**Figure S7** | Computed harmonic phonons as a function of pressure	**S14**
**Table S2** | Computed elastic tensor at 20 GPa and stability criteria	**S14**
**Figure S8** | Rietveld refinement of K_5Ir model: Graphical results	**S15**
**Table S3** | Rietveld refinement of K_5Ir model: Crystallographic data	**S16**
**Table S4** | Rietveld refinement of K_5Ir model: Atomic coordinates	**S16**
**Table S5** | Rietveld refinement of K_5Ir model: Comparison with DFT-optimized structure	**S16**
**Figure S9** | Identifying the Ir 6*s* band	**S17**
**Table S6** | Net atomic charges: Pressure-dependence of phases studied with Ir L_3 XANES	**S17**
**Figure S10** | Computed charge density close to the Fermi energy on the $(11\bar{2}0)$ plane	**S18**
**Figure S11** | Computed electron localization functions (ELFs) for K_5Ir and *bcc*-K	**S18**
**Table S7** | Net atomic charges: Alkali−5*d* and alkali−3*p* insulators and comparison to K_5Ir	**S19**
**Table S8** | Net atomic charges: Comparison of methods and levels of theory	**S19**
**Table S9** | Net atomic charges: Pressure-dependence of alkali–5*d* insulators and K_5Ir	**S19**
**Table S10** | Net atomic charges: Reduced transition metal carbonyl complexes	**S20**
**Table S11** | Net atomic charges: Reported alkali- and alkaline earth-iridium intermetallics	**S20**
**Figure S12** | Crystal structure prediction for the K–Ir system at zero pressure	**S21**
**Figure S13** | Computed thermodynamics of K5Ir formation between 0 and 20 GPa	**S22**
**References**	**S23**

Experimental Details

Starting materials and diamond anvil cell loading

CAUTION: Potassium metal reacts violently with water and forms reactive peroxides in air. Appropriate measures must be taken for safe handling, cleaning, and disposal. Potassium (99%, ThermoFisher Scientific: 9800 ppm Na, 44 ppm Ca, < 1 ppm other metals by manufacturer analysis) and Ir powder (-60 mesh, 99.95%, Alfa Aesar: < 300 ppm other metals by manufacturer analysis) were purchased and used without further purification. However, iridium powder was sieved to -325 mesh in a nitrogen glovebox before use. All samples were pressurized in BX-80 diamond anvil cells (DAC Tools LLC) equipped with 300 μm culet diamonds (Almax easyLab Inc.) and tungsten carbide seats (Almax easyLab Inc.). Gaskets were pre-indented from 250 μm rhenium sheet (H. Cross Company) to a thickness of 20 μm to 32 μm (preliminary, thicker samples led to blowouts or extrusion of K from the sample space during compression), laser-drilled to form 150 μm diameter cylindrical sample spaces, and sonicated with isopropyl alcohol. Iridium was loaded into the sample space in air (except for the sample with KBr described below, which was loaded in a nitrogen glovebox) either as loose particles or pre-pressed between the diamonds into a fragile aggregate (iridium is not malleable enough to form a robust foil by this method). Subsequently, freshly cut potassium was loaded in a nitrogen glovebox by spreading a small quantity on the culet of the top diamond and closing the DAC such that excess potassium is extruded out of the sample space via the contact between the pavilion of the diamond and the gasket. Given this loading procedure, the precise K:Ir ratios are not known, but are estimated to be quite K-rich in all cases based on the approximate volumes of Ir loaded and the initial sample space volumes. Samples prepared in this way are air-tight at least on the timescale of many months so long as they are pressurized to ∼2 GPa before removal from the glovebox.

Laser-heating experiments

For laser-heated samples, single crystal KBr and MgO substrates were glued to microscope slides and sanded by hand (for KBr, in a nitrogen glovebox) to an approximate thickness of 20 μm. MgO disks were laser drilled in air, while KBr pieces were cleaved mechanically with a needle in a nitrogen glovebox. These were loaded into the sample space on one side prior to the iridium to serve as thermal insulators. Single-sided laser heating was performed on beamline 13-ID-D of the GSECARS user facility at the Advanced Photon Source (Argonne National Laboratory), which has been described previously.[1] Sample temperature was estimated by fitting emission from the sample to a blackbody spectrum using T-Rax 1.4.2 (https://github.com/CPrescher/T-Rax). For the sample depicted in Figure S1, the laser power was incremented until apparent iridium melting (based both on the calculated fusion curve[2] and on visual indicators) and the laser spot was subsequently rastered over a significant portion of the sample space, adjusting laser power as necessary to sustain the temperature through changes in coupling to the sample.

Whole-cell heating experiments

Whole-cell heating was conducted under rough dynamic vacuum in a Shel Lab drying oven equipped with a Watlow temperature controller. Samples were heated from room temperature to 493 K in 3 h, held for either 15 h or 150 h, and cooled to room temperature in 3 h. This reaction temperature stays below the maximum constant working temperature for the J-B Weld epoxy joining the diamonds and the seats. Despite our expectation that creep would lead to pressure reduction during prolonged heating, we instead observed slight pressure increases between pre- and post-heating measurements.

Raman spectroscopy

Raman spectra were recorded with 532 nm incident light using the Laser Lab spectrometer of the GSECARS user facility at the Advanced Photon Source (Argonne National Laboratory), which has been described previously.[3] Spectra were processed using T-Rax 1.4.2 and pressures were assigned based on the reported scale for the high-shift Raman edge of diamond.[4]

X-ray diffraction

Laboratory X-ray diffraction was recorded on a STOE Stadivari diffractometer equipped with a sealed tube Ag source, an Eulerian cradle with a diamond anvil cell attachment, and a DECTRIS EIGER2 R CdTe 1M detector. Synchrotron X-ray imaging and microdiffraction was performed at beamline 16-ID-B of the HPCAT user facility at the Advanced Photon Source, Argonne National Laboratory, using a photon energy of 29.2 keV. The nominal microfocussed spot is 2 μm × 2 μm. Masking, integration, and some analyses were performed with DIOPTAS.[5] Cell indexing and Rietveld refinement were performed with GSAS-II.[6] For Rietveld refinement, the background was fit with a 14-term Chebyschev polynomial and 4th-order spherical harmonics were used to approximate the preferred orientations of the few-domain excess K and the textured Re gasket. Other than slight influence on ADPs as would be expected, results were found to be insensitive to the omission or inclusion of a fixed absorption correction based on K at the appropriate density, which dominates the path length for photons through the sample.

X-ray absorption spectroscopy

Iridium L_3-edge X-ray absorption spectroscopy in the near-edge region (XANES) was performed on beamline BM23 at the European Synchrotron Radiation Facility, which has been described previously.[7] These experiments used a Si(111) monochromator, harmonic rejection via Pt-coated mirrors inclined to 4.5 mrad, ion chambers mounted before and after the sample, and a microfocussed 3 μm × 3 μm beam. An absorption map at 11.5 keV was recorded with a 2 μm step size. Ir metal foil (as energy calibrant) and an IrO_2 pellet were measured at ambient pressure. The DAC sample containing K_5Ir was recorded at 19.5(6) GPa. Spectra were recorded at different rotation angles of the DAC to determine experimental conditions where artifacts from the diamonds do not fall close to the Ir L_3-edge. Spectra recorded away from the K_5Ir needles in the excess K phase (*fcc*) show no detectable alloying of Ir. Spectra recorded on a grid are homogeneous other than varying strength of the K_5Ir signal, indicating no detectable Ir-containing phases other than K_5Ir. For presentation and model fitting, each spectrum in Figure 4a was normalized in a two-step procedure: 1) A linear background, fit below the absorption edge, was subtracted; 2) The resulting spectrum was scaled so that the average of the oscillatory near-edge region above the prominent $2p_{3/2} \rightarrow 5d$ absorption was 1.

Computational Details

Density functional theory (DFT) and hybrid functional calculations were performed using VASP 6.4.2.[8–11] The VASP-provided projector augmented wave (PAW) PBE pseudopotentials[12,13] from the 6.4 release (those labeled K sv, Cs sv, Li sv, Mg, Ca sv, Au, Pt, Ir, Re, P, S, Cl, C h, O h) were used except for the DDEC6 calculations, which used those from the 5.4 release for code compatibility.

Functionals

Except as noted in the sensitivity analysis described in Table S1 below, the PBE generalized gradient approximation (GGA) functional[14] was used for computing self-consistent structures, phonons, elastic moduli, and formation enthalpies. Screened hybrid functional calculations used the HSE06 functional (25% Fock exchange at short-range, 0.2 $Å^{-1}$ range separation, PBE for the DFT part)[15] to improve the description of the electronic structure.[16]

Technical parameters

All calculations employed a Fourier mesh with $2 \times G_{cut}$ points along each lattice vector, while local augmentation charges were represented on a mesh of twice this linear density. The spin orbit interaction (SOC)[17,18] was included in describing the electronic band structure, density of states, and Fermi surface of K_5Ir. Other calculations included only the scalar relativistic terms. Excepting the crystal structure prediction described below, calculations used a plane wave basis set cutoff energy of 250 eV (340 eV for CsCl and Cs_2S, 350 eV for Ca_5Ir, 650 eV for LiIr, $LiIr_3$, and Li_2MgIr, 700 eV for the carbonyl complexes due to the hard C and O pseudopotentials) and *k*-mesh densities such that $N_k \times N_{atom} > 500$, based on convergence of the total energy to within 10 meV $atom^{-1}$ of the apparent asymptotic limit.

Wannierization and visualization of the Fermi surface

To compute the eigenvalues at the HSE+SOC level on fine meshes (DOS, Fermi surface) or paths (band structure), maximally-localized Wannier functions were constructed[19] from entangled energy bands[20] using Wannier90.[21] Fermi surfaces were visualized using the py_FS code (https://github.com/TheoWeinberger/py_FS).[22]

Net atomic charges and other real-space electronic structure quantities

Structures for the net atomic charge comparison were taken from the relevant reports.[23–37] The structure of $Na_3[Ir(CO)_3]$ has not been reported, but the complex is putatively trigonal planar based on its infrared spectrum and on structurally-characterized derivative complexes.[32] Isolation of $[Re(CO)_4]^{3-}$ from solvent has not been reported, but the complex is presumed tetrahedral based on its infrared spectrum.[29] Therefore, these complexes were studied by placing the isolated complex at the vertices of a large unit cell (Ir: a = c = 10 Å, $P\bar{6}2m$ resulting symmetry; Re: a = 10 Å, $P\bar{4}3m$ resulting symmetry) and applying a homogeneous neutralizing background jellium. DDEC6 net atomic charges were computed using chargemol (https://sourceforge.net/projects/ddec/files/).[38,39] Bader charges were computed using the code of the Henkelman group (https://theory.cm.utexas.edu/henkelman/code/bader).[40–43] Löwdin charges were computed using LOBSTER.[44,45] Electron localization functions[46–48] were computed using VASP's internal routine. DOS orbital projections without spin-orbit coupling were computed with LOBSTER.[44,45]

Mechanical properties

Interatomic force constants were computed by density functional perturbation theory as implemented in VASP and the resulting harmonic phonons and their irreducible representations were determined using phonopy.[49,50] The elastic tensor was computed by the stress-strain method using finite differences as

implemented in VASP and was tested against the stability criteria of Mouhat and Coudert for hexagonal crystals.[51]

Note on the energy-restricted partial charge density

The partial charge density near E_F was computed without the use of symmetry internally in VASP. This ensures that the density reflects the true symmetry, rather than (for each band which falls within the energy window) corresponding only to the single representative wavevector of the star which falls in the irreducible Brillouin zone. The large number of *k*-points necessary for this approach rendered hybrid functionals impractical for this purpose, so the PBE functional was used.

Post hoc crystal structure prediction

Crystal structure prediction was performed using the USPEX code based on an evolutionary algorithm developed by Oganov, Glass, Lyakhov and Zhu and featuring local optimization, real-space representation and flexible physically motivated variation operators.[52–54] The evolutionary algorithm was run for a total of 40 generations, with 30 individuals in the first generation and 20 individuals in subsequent generations. The first generation was initiated with 11 compositions ranging from 4 to 12 atoms. For each individual at each generation, six sequential DFT calculations with increasingly rigorous parameters were performed, to a final cutoff energy of 450 eV, a final force criterion of 4 meV $Å^{-1}$, and a final *k*-spacing of < 0.08 $Å^{-1}$.

Other software tools

Crystal structures, valence charge densities, and ELFs were visualized using VESTA.[55] Analysis of candidate structures utilized pymatgen,[56] spglib,[57] and the SAM program of the Bilbao Crystallographic Server.[58]

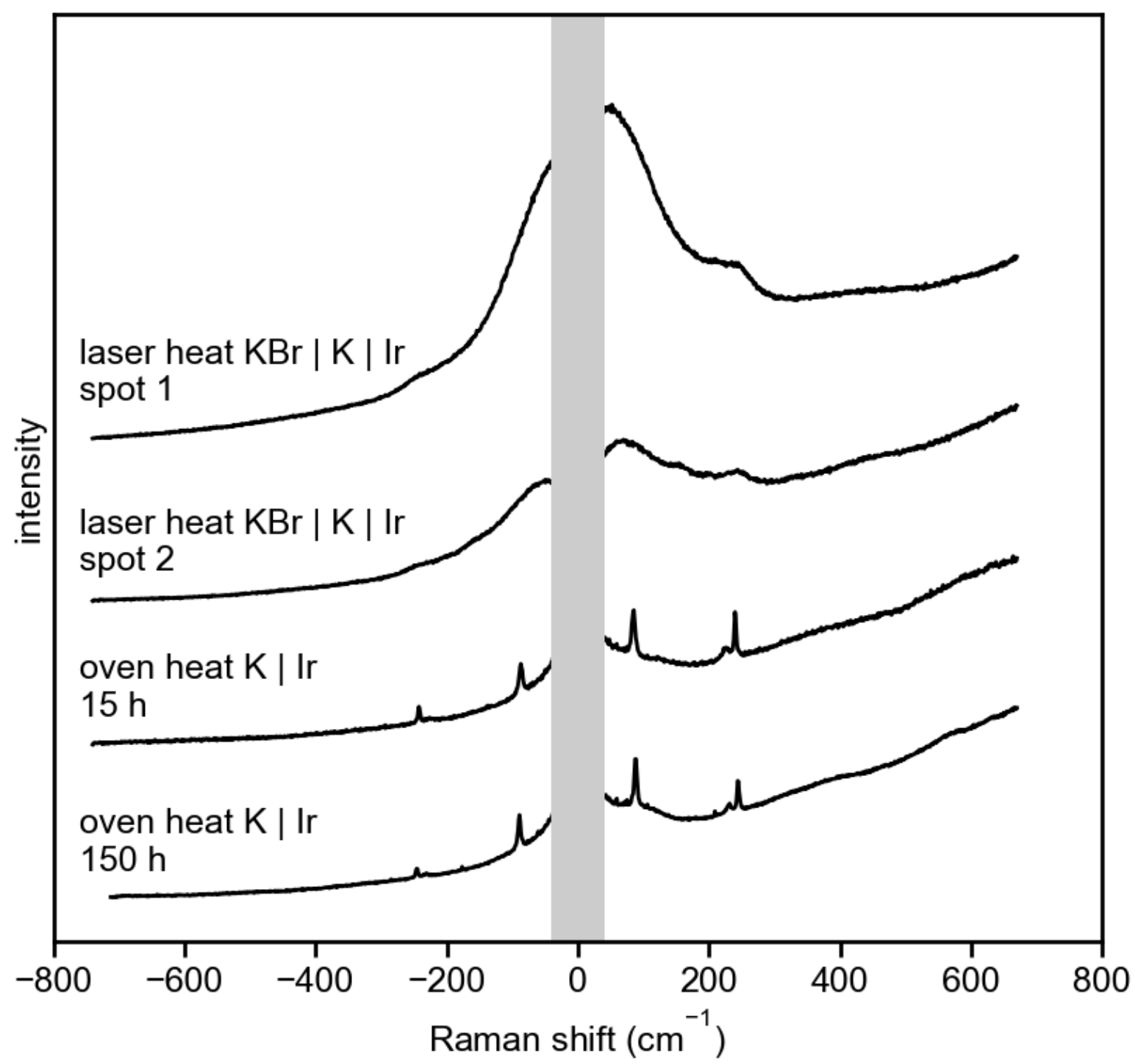

Figure S1 | Raman spectroscopy from a sample laser-heated from one side with a KBr flake as a thermal insulator shows broad Raman scattering features covering the same range of frequencies as the sharp modes present in oven-heated samples. Based on diamond Raman, the pressure in the laser-heated sample was 20.5(15) GPa across the cell, compared to 17.7(1) GPa for the 15 h oven-heated sample and 19.5(6) GPa for the 150 h oven-heated sample.

Table S1 | After initial reactions did not exhibit clear signs of reactivity between K and Ir, we carried out a sensitivity analysis of the formation enthalpy (ΔH_f) of K_3Ir (the most stable phase proposed by Brgoch and Hermus, space group *Pmmn*)[37] at 20 GPa. Structures of K_3Ir and the elements were relaxed self-consistently for each of these cases. "Larger basis set, finer k-mesh" refers to E_{cut} = 400 eV, Nk × Natom > 1000. "Include K-III" uses an 8/5 approximant to the incommensurate host−guest structure of K-III,[59] while the other configurations use the structure of K-II (*fcc*).[60] None of these technical enhancements materially impacted the conclusion that reaction of K and Ir should be exothermic at zero temperature. The magnitude of the formation enthalpy exceeds typical differences in the finite temperature terms between solid phases without configurational disorder, suggesting experimental or kinetic challenges as the origin of the initial apparent lack of reactivity.

**Case**	**ΔH_f (eV atom^{-1})**	**$\Delta H_f - \Delta H_f\|_{\mathrm{baseline}}$ (meV atom^{-1})**
baseline	−0.386	+0
larger basis set, finer k-mesh	−0.387	−1
PBEsol	−0.345	+41
PBE+SOC	−0.352	+34
HSE06	−0.439	−53
include K-III	−0.373	+13

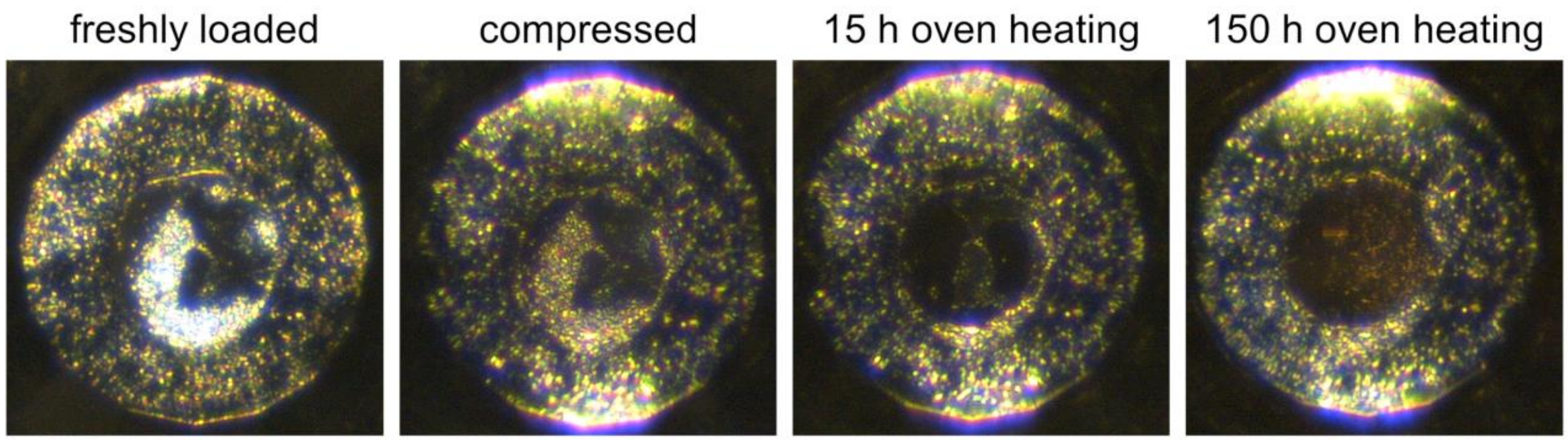

Figure S2 | Optical microscopy at key steps of sample processing. All micrographs are taken on the piston side of a DAC loaded with Ir and K and lit from above. The sample diameter is 150 µm before compression. No light transmission is visible through the sample space when backlit at any stage of processing (not shown). Upon loading (∼3 GPa), black iridium powder aggregates and silver-colored potassium are observed. Upon compression (∼19 GPa), the potassium reflectivity in the visible is reduced, in accordance with prior reports.[61,62] After 15 h oven heating, the potassium/diamond interface appears smooth, presumably from potassium melting and re-solidification. Successful formation of a potassium melt also explains the spatial redistribution of black iridium particles. After 150 h oven heating, there are dramatic changes in the sample appearance, as shown in more detail in Figure 1(a), including the absence of black regions resembling the iridium powder precursor.

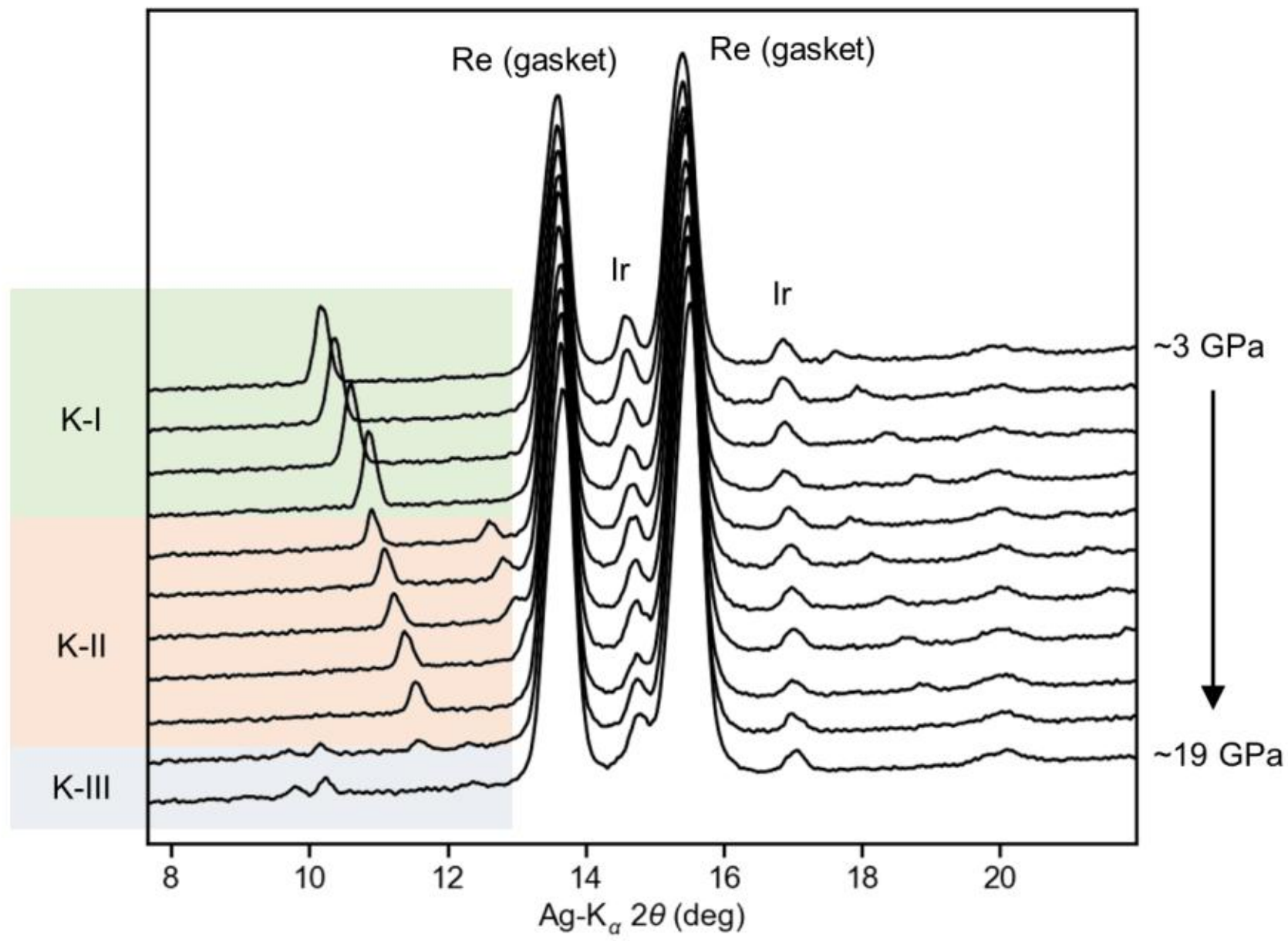

Figure S3 | Azimuthally-integrated laboratory X-ray diffraction from a precursor mixture of elemental K and Ir during compression to ∼19 GPa, before heating. The most prominent reflections from the Re gasket and the Ir are annotated as well as the pressure regions of the various K polymorphs. As noted by others, most reflections from the incommensurate K-III phase are weak upon cold compression.

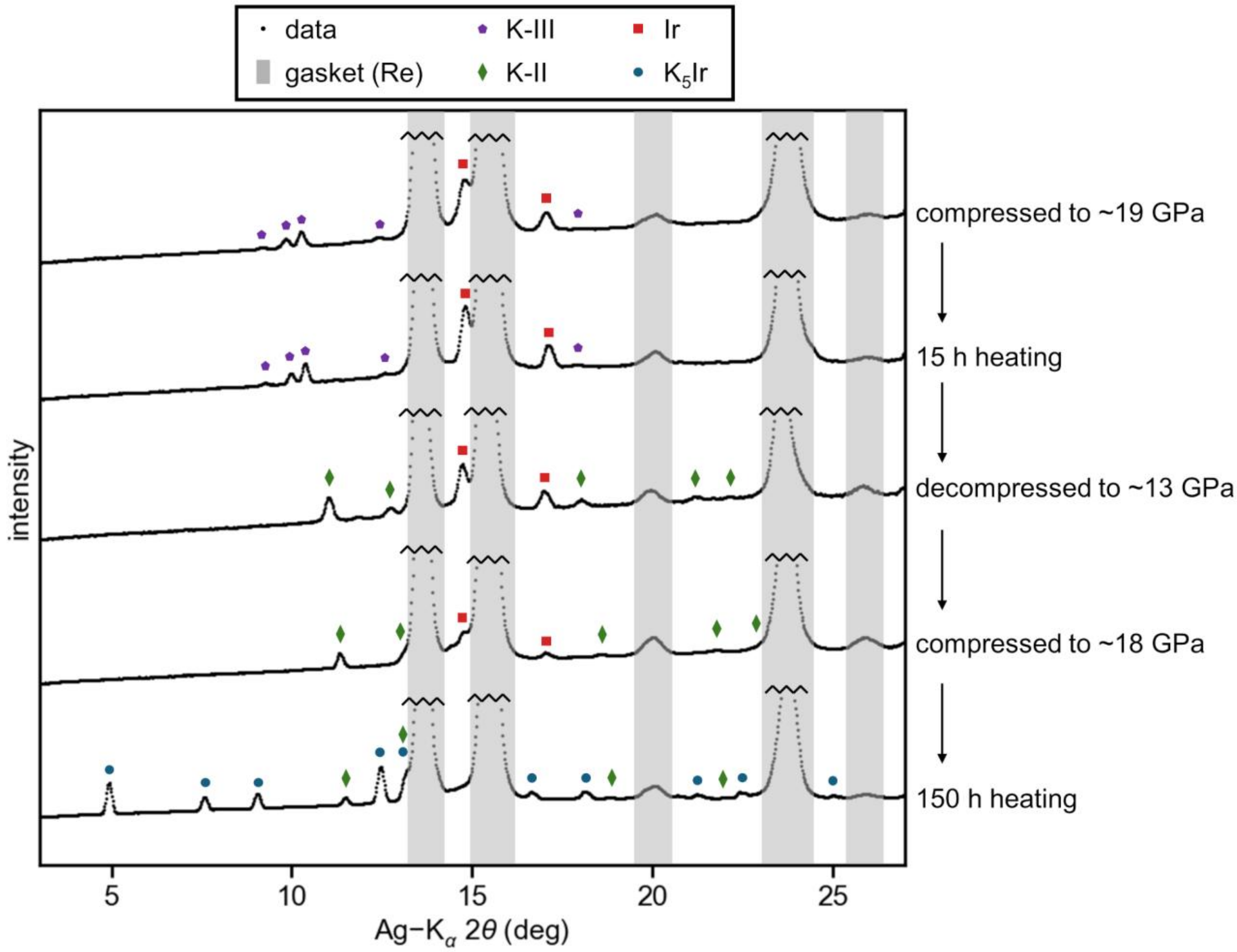

Figure S4 | Azimuthally-integrated laboratory X-ray diffraction (Ag−K_α, ~150 μm ∅ beam) from a sample of K and Ir at key processing steps. Pressures annotated here are based on the reported equation of state for Ir.[2] Bragg reflections from the relevant phases at each step are annotated. The strongest reflections from the textured Re gasket have been clipped for clarity.

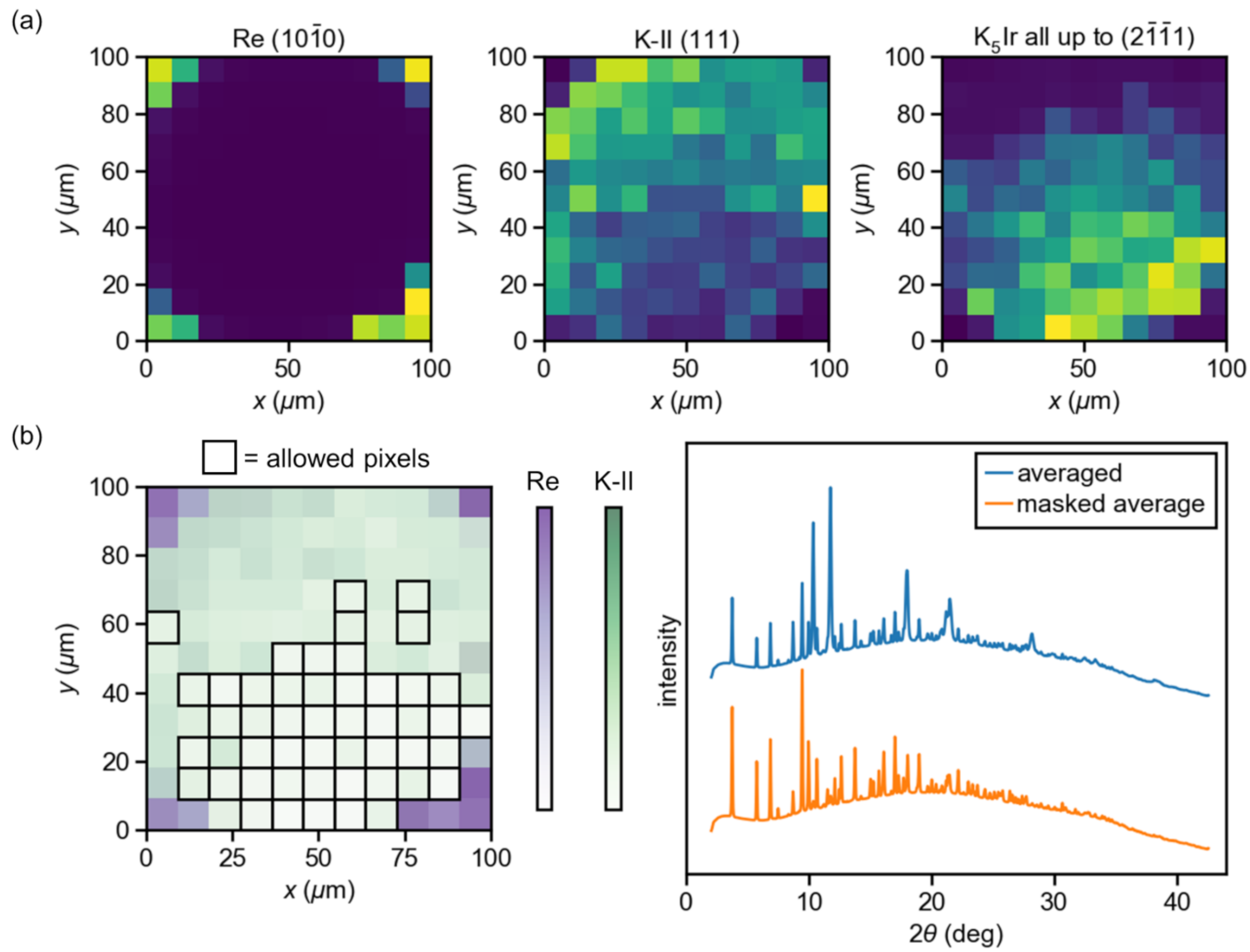

Figure S5 | Spatial distribution of phases at 19.5(6) GPa, as determined by the intensity of Bragg reflections observed by synchrotron microdiffraction. (a) The rhenium gasket is evident at the corners of this grid scan, while the inhomogeneous distribution of K-II and K_5Ir matches the expectations from optical microscopy and X-ray imaging in Figure 1(a-b). Specifically, K_5Ir is found where the dark needles are visible and the X-ray transmission is low, while K-II is most prevalent elsewhere. (b) Spatial averaging of microdiffraction was performed over a masked subset of pixels based on the Re and K-II contributions. This masked averaging reduces shape and orientation effects on the K_5Ir Bragg intensities while minimizing peak overlap with the strained and textured gasket reflections and the strongly oriented excess K reflections.

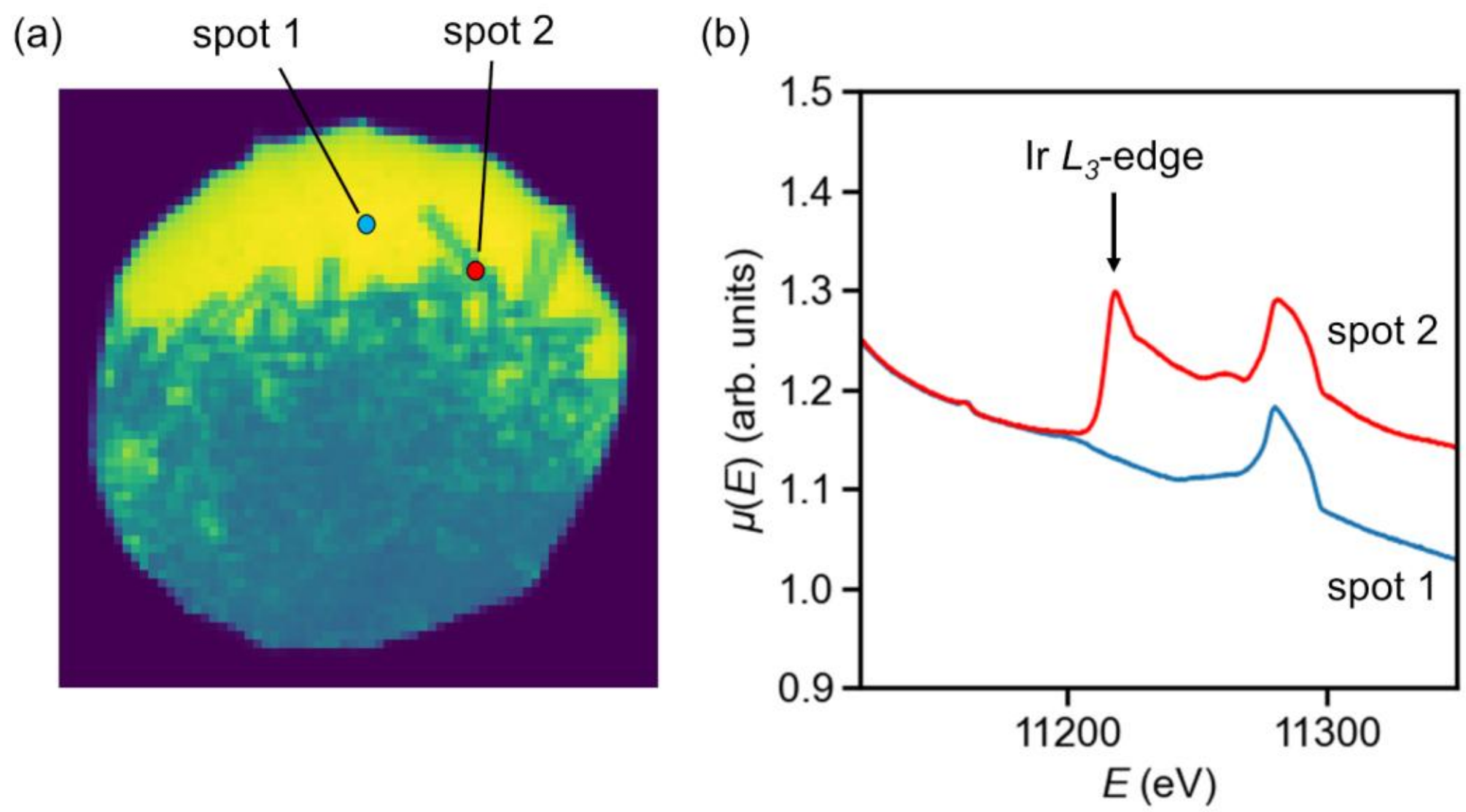

Figure S6 | Iridium L_3-edge X-ray absorption spectra from two different microfocussed spots at 19.5(6) GPa. (a) Absorption map at 11.5 keV. (b) Spectra vertically aligned to match in pre-edge region ("arb. units" = arbitrary units). The spectrum recorded in the matrix phase ("spot 1") shows no detectable signal at the Ir L_3-edge, suggesting negligible alloying of Ir into the excess K phase. The features peaked around 11.28 keV are diamond artifacts.

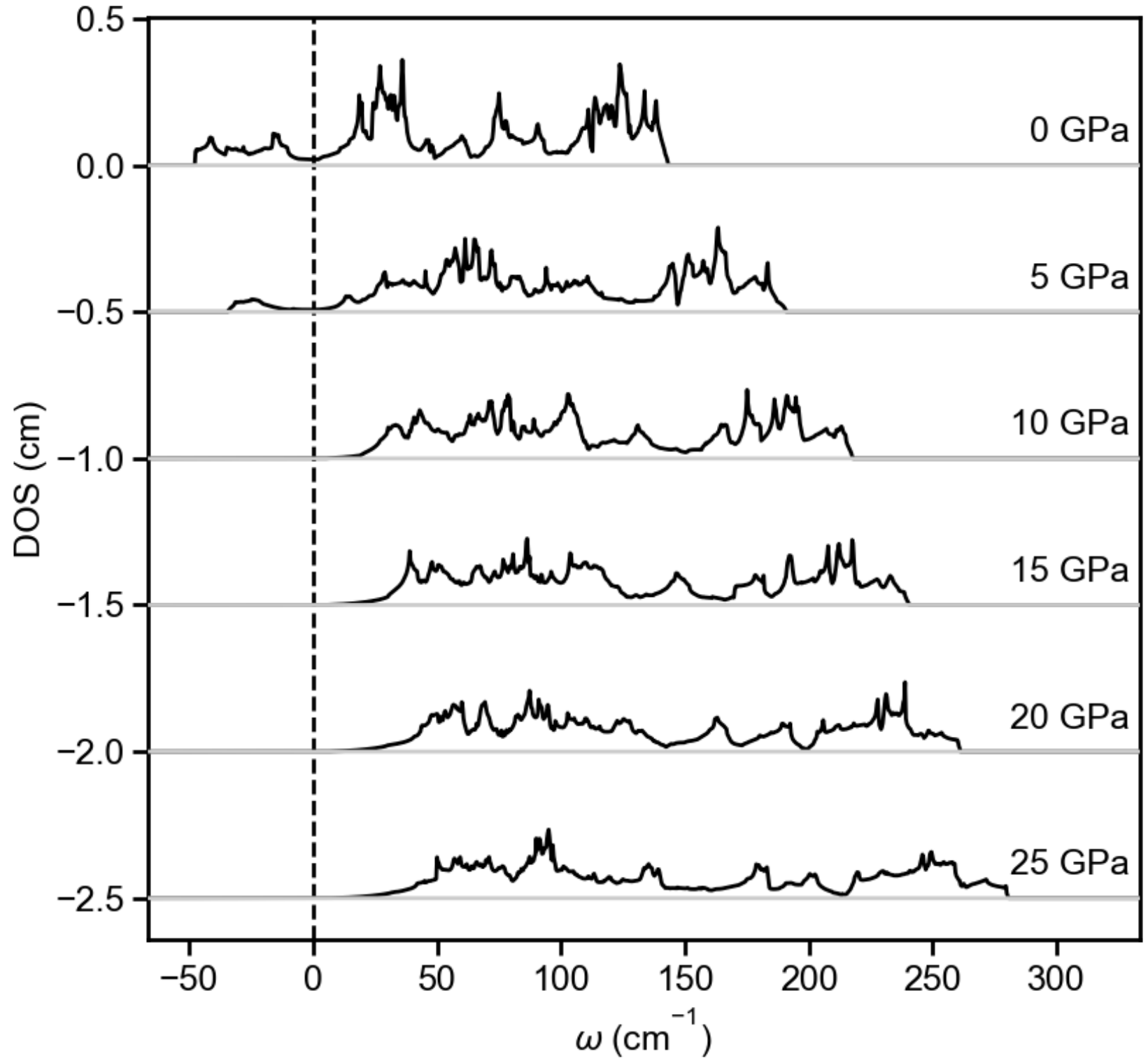

Figure S7 | Harmonic phonon calculations (PBE) indicate dynamic stability of K_5Ir at 10 GPa, 15 GPa, 20 GPa, and 25 GPa but dynamic instability at 0 GPa and 5 GPa. Successive curves are offset as indicated by the gray lines.

Table S2 | The elastic moduli computed at 20 GPa by the stress−strain method (PBE) meet the criteria for elastic stability of hexagonal crystals.[51]

	C_{11}	C_{12}	C_{13}	C_{33}	C_{44}	C_{66}
value (GPa)	86.8	44.3	30.0	92.9	12.4	21.2

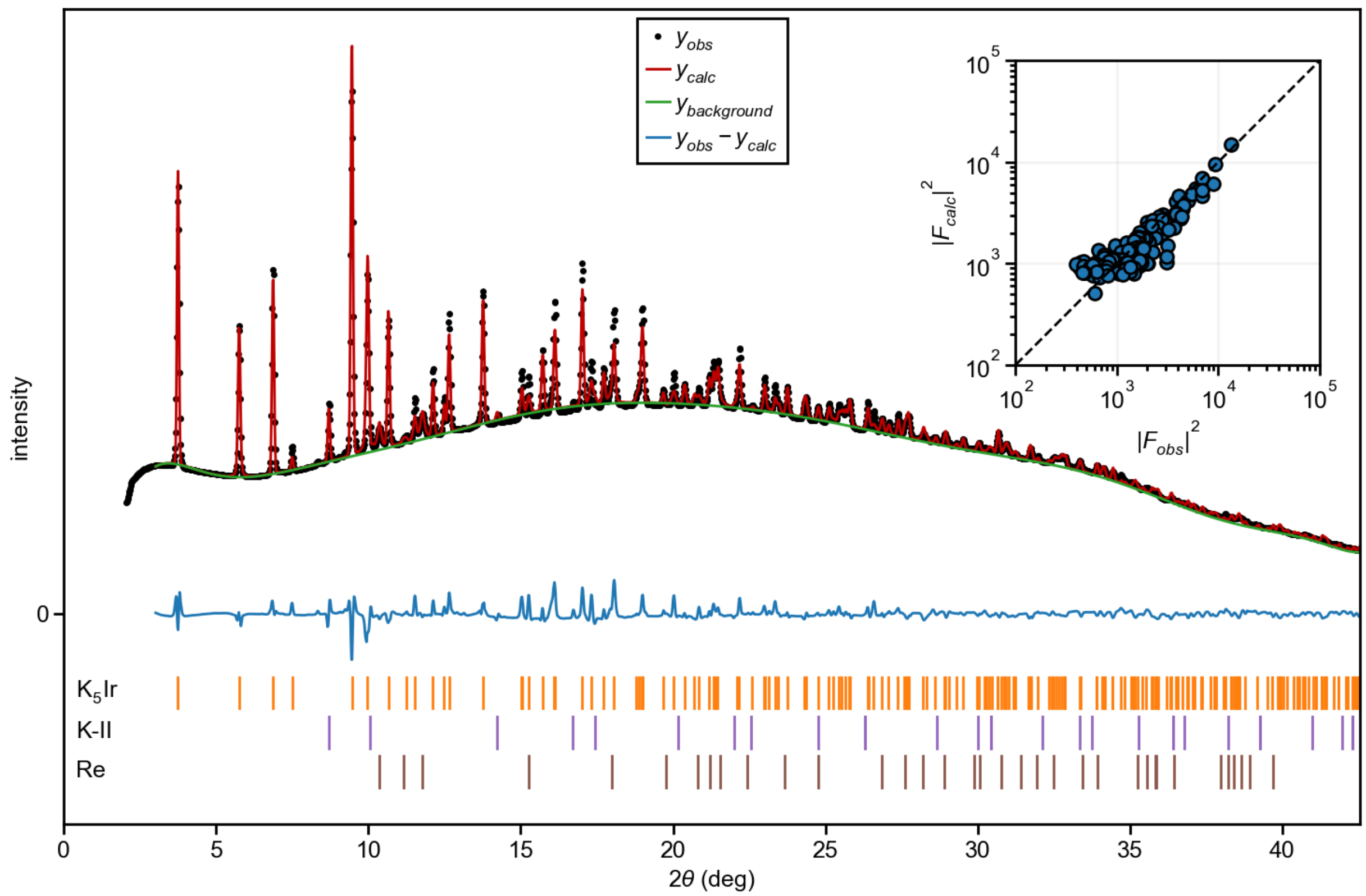

Figure S8 | Graphical results of Rietveld refinement at 19.5(6) GPa, from synchrotron diffraction at $\lambda = 0.42459$ Å (16-ID-B, Advanced Photon Source). These high pressure data on an ensemble of crystallites present challenges relative to typical ambient pressure diffraction from powders, in the form of a strong and complex background, imperfect orientation averaging, texture of the Re gasket and excess K, and imperfect fitting of the peak profile. The atomic displacement parameters (ADPs), in particular, should be interpreted with care. The only free atom position parameter, $z_{K(4h)}$, is robust to refinement strategy and is corroborated by DFT (Table S5). Bragg reflections from the three phases present in the model are indicated with ticks. A comparison of observed and modeled structure factors for K_5Ir is inset.

Table S3 | Crystallographic data for K_5Ir at room temperature and 19.5(6) GPa from Rietveld refinement of synchrotron X-ray diffraction. (*) As defined in GSAS-II.

sum formula	K_5Ir
molecular weight (g mol^{-1})	387.709
temperature (K)	293
pressure (GPa)	19.5(6)
space group	*P*6/*mmm* (#191)
Z	1
a (Å)	4.8863(4)
c (Å)	6.49897(31)
V (Å^3)	134.379(11)
ρ_{calc} (g cm^{-3})	4.7910
wavelength (Å)	0.42459
R_p (%)*	1.558
R_{wp} (%)*	2.325
R_{exp} (%)*	1.529
G.O.F.*	1.53
no. of variables	26

Table S4 | Atomic coordinates for K_5Ir at room temperature and 19.5(6) GPa from Rietveld refinement of synchrotron X-ray diffraction. Given the data limitations associated with diffraction from a collection of oriented crystallites at high pressure described in the caption of Figure S7, the ADPs should be interpreted with care.

Atom	Wyckoff label	Site symmetry	*x* / *a*	*y* / *b*	*z* / *c*	U_{iso} (Å^2)
K1	1a	6/*mmm*	0	0	0	0.072(5)
K2	4h	3*m*.	1/3	2/3	0.2911(7)	0.0449(24)
Ir1	1b	6/*mmm*	0	0	1/2	0.0016(4)

Table S5 | Comparison of Rietveld-refined and DFT-optimized structures for K_5Ir.

	Experiment 19.5(6) GPa	DFT (PBE) 20 GPa	(DFT−Expt.) / Expt.
a (Å)	4.8863(4)	4.8688	−0.4 %
c (Å)	6.49897(31)	6.37896	−1.8 %
$z_{K(4h)}$ / *c*	0.2911(7)	0.2798	−3.9 %

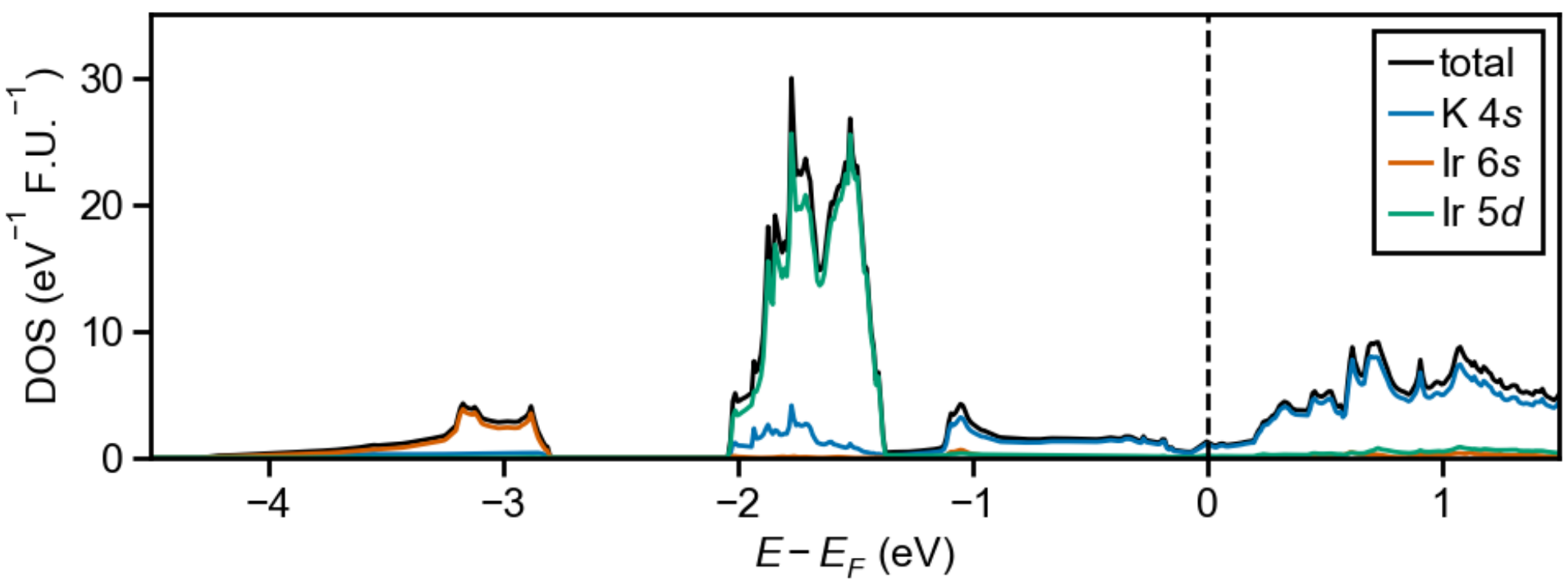

Figure S9 | The projected density-of-states at the PBE level for K_5Ir at 20 GPa allows one to identify the states deriving from the Ir 6*s* orbitals. The relative energetic ordering of Ir 6*s* and 5*d* bands supports the anionic character of Ir in K_5Ir.[63] As of writing, VASP does not output the cell-periodic part of the Bloch functions in a manner that is compatible with Wannier90 for noncollinear calculations, preventing the direct visualization of Wannier orbitals constructed from the hybrid functional calculations with spin-orbit coupling (HSE+SOC). The primary effect of SOC will be to split the Ir $5d_{3/2}$ and $5d_{5/2}$ states, leading to the separated sets of bands in Figure 3. Comparison of these results with those from Wannierization of the HSE+SOC band structure identifies the band between −3 eV and −5 eV in Figure 3 as the Ir 6*s* band. We note also that the dispersion of this band on the path Γ–M–K–Γ in Figure 3 looks like a modest perturbation of the dispersion for a hexagonal lattice of *s* orbitals interacting via first nearest neighbor hopping, further supporting this assignment.

Table S6 | Computed pressure dependence of Ir DDEC6 net atomic charges at the HSE level for the phases studied experimentally with Ir L_3-edge XANES. Net atomic charges for unary Ir are 0 by definition. Ir net atomic charges in IrO_2 are not affected by pressure across this range, and those in K_5Ir are only weakly affected.

**phase**	**0 GPa**	**20 GPa**
Ir	0	0
IrO_2	+1.38	+1.38
K_5Ir	–1.84	–1.89

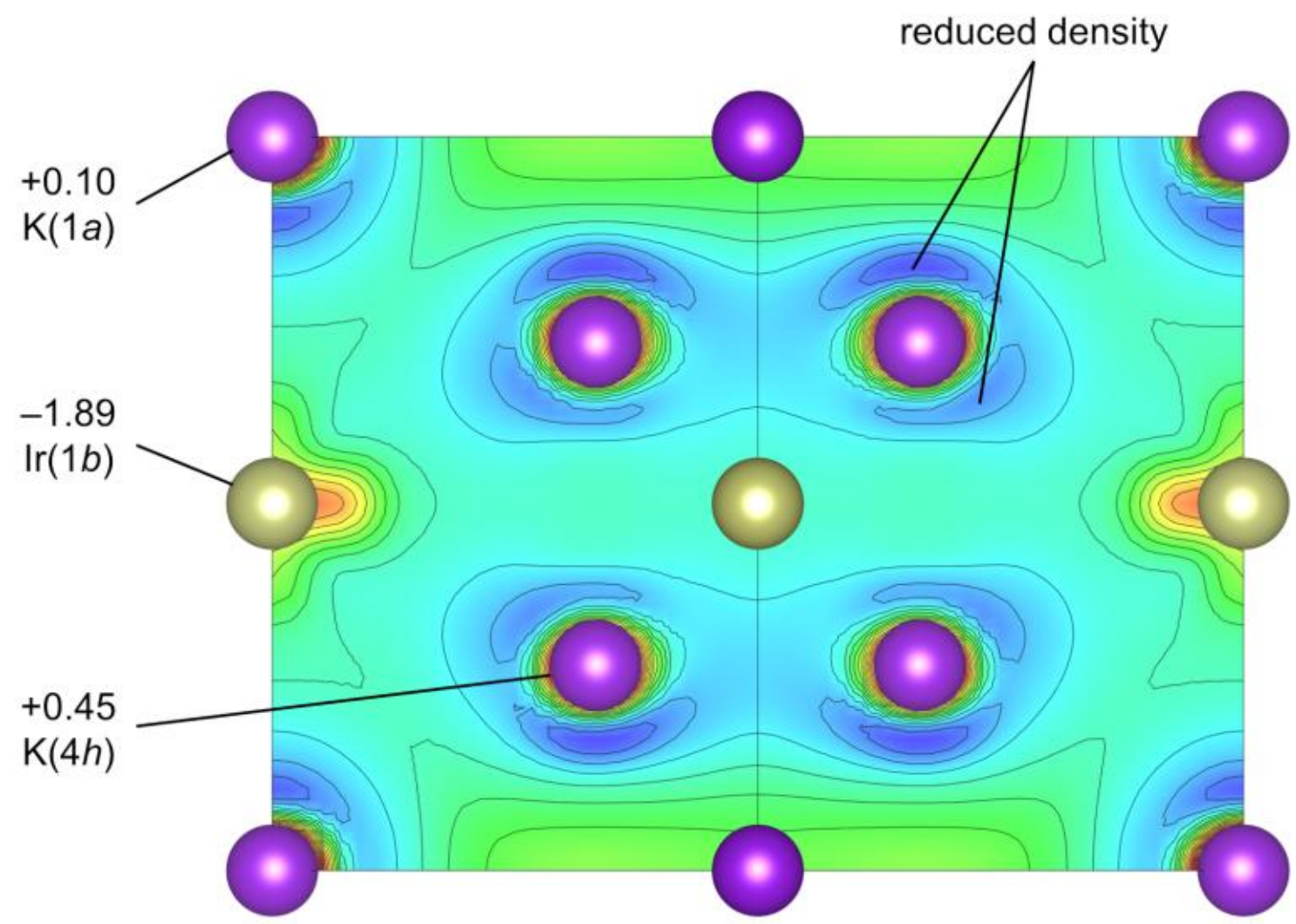

Figure S10 | Valence charge density (DFT–PBE, see "*Note on the energy-restricted partial charge density"*) within $E_F \pm 100$ meV on the $(11\bar{2}0)$ plane and site-specific DDEC6 net atomic charges (HSE) for K_5Ir at 20 GPa. In contrast to the weakly elevated charge density bridging the (0001) planes containing the neutral K (1*a* site) via the Ir sites, the charge density exhibits local minima between the cationic K (4*h* site) and both the Ir and the (0001) planes containing neutral K.

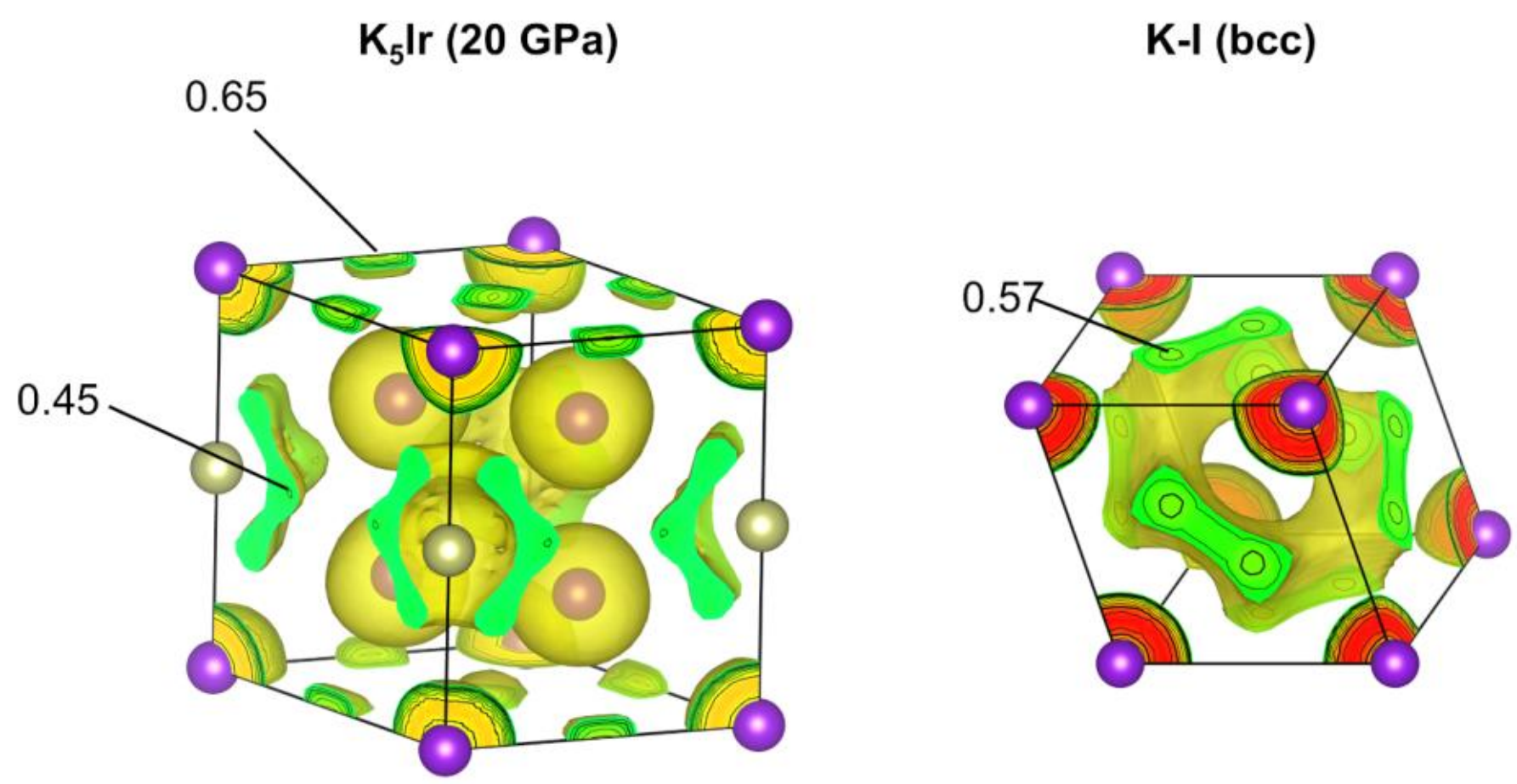

Figure S11 | Electron localization functions computed at the HSE level, with isosurfaces depicted at ELF = 0.40 and values at selected local maxima annotated. The ELF for K_5Ir shows covalency features between atoms similar to those in elemental metals like *bcc*-K, particularly in the (0001) planes containing the neutral K (1*a* site). In contrast, ionic semiconductors CsAu, Cs_2Pt, and hypothetical *Pmmn* K_3Ir (20 GPa) show only core-like ELF attractors, reflecting complete charge transfer from cations to anions.

Table S7 | DDEC6 net atomic charges ("N.A.C.") at the HSE level for the anions in insulating alkali−5*d* and alkali−3*p* phases, with comparison to that in K_5Ir. (*) K_3Ir refers to the hypothetical *Pmmn* phase predicted by Brgoch and Hermus from crystal structure prediction at a fixed composition.[37]

**oxidation state**	**alkali−5*d***		**alkali−3*p***	
	**phase**	**N.A.C.**	**phase**	**N.A.C.**
(I-)	CsAu	−0.81	CsCl	−0.86
(II-)	Cs_2Pt	−1.45	Cs_2S	−1.54
(III-)	K_3Ir(*), 20 GPa	−2.13	K_3P	−2.05
n/a (semimetal)	K_5Ir, 20 GPa	−1.89		

Table S8 | Comparison of net atomic charges computed by different methods and at different levels of theory. (*) K_3Ir refers to the hypothetical *Pmmn* phase predicted by Brgoch and Hermus from crystal structure prediction at a fixed composition.[37] All three atomic charge types tested here give qualitatively similar results other than the relative ordering of K_3Ir and K_5Ir.

	**DDEC6 (HSE)**	**DDEC6 (PBE)**	**Bader (PBE)**	**Löwdin (PBE)**
CsAu	−0.81	−0.79	−0.76	−0.66
Cs_2Pt	−1.45	−1.37	−1.31	−1.42
K_3Ir(*), 20 GPa	−2.13	−1.99	−1.69	−1.89
K_5Ir, 20 GPa	−1.89	−1.79	−1.91	−2.16

Table S9 | Computed pressure dependence of transition metal DDEC6 net atomic charges at the HSE level for alkali metal–5*d* semiconductors and for K_5Ir. (*) K_3Ir refers to the hypothetical *Pmmn* phase predicted by Brgoch and Hermus from crystal structure prediction at a fixed composition.[37] Across this range, the pressure dependence of these phases is weak.

**phase**	**0 GPa**	**20 GPa**
CsAu	−0.81	−0.80
Cs_2Pt	–1.45	–1.34
K_3Ir(*)	–1.97	–2.13
K_5Ir	–1.84	–1.89

Table S10 | DDEC6 net atomic charges ("N.A.C.") at the HSE level for selected anionic transition metal carbonyl complexes. Isolation of $[Re(CO)_4]^{3-}$ from the solvent has not been reported, nor has a structure for $Na_3[Ir(CO)_3]$ been reported, so we consider the putative tetrahedral and trigonal planar complexes, respectively, which are postulated based on their infrared (IR) spectra.[29,32] Consistent with expectations for strong-field CO ligands, despite the high negative oxidation state (III-), the net atomic charges of Ir and Re in these complexes are well shy of those of Pt^{2-} in Cs_2Pt, reduced Ir in K_5Ir, and Ir^{3-} in hypothetical K_3Ir. The group 6 metals with oxidation state (IV-) in $Na_4[M(CO)_4]$ (M = Cr, Mo, W) cannot be similarly examined because their IR spectra suggest more complex unknown structures.[64]

oxidation state	phase	N.A.C.
(III-)	$[Ir(CO)_3]^{3-}$	−1.16 (Ir)
(III-)	$[Re(CO)_4]^{3-}$	−1.24 (Re)

Table S11 | DDEC6 net atomic charges ("N.A.C.") at the HSE level for the the iridium in LiIr and in alkali- and/or alkaline earth-rich intermetallics. The multiple values for $Mg_{29}Ir_4$ and $Mg_{44}Ir_7$ correspond to the distinct Ir sites. The ratio of the sum of the number of alkali metal atoms (n_A) plus twice the number of alkaline earth metal atoms (n_E) to the number of iridium atoms (n_{Ir}), a naive measure of the number of electrons available to reduce each iridium in the limit of complete ionization of the *s*-elements to the group valence, is also given.

phase	N.A.C.	$(n_A + 2n_E) / n_{Ir}$
K_5Ir, 20 GPa	−1.89	5.0
LiIr	−0.67	1.0
Li_2MgIr	−2.23	4.0
Ca_5Ir	−1.89	10.
$Mg_{29}Ir_4$	−2.28, −2.13, −2.02	14.5
$Mg_{44}Ir_7$	−2.20, −2.18, −2.15	12.6
Mg_3Ir	−2.36	6.0
$Mg_{13}Ir_3$	−2.35	8.7

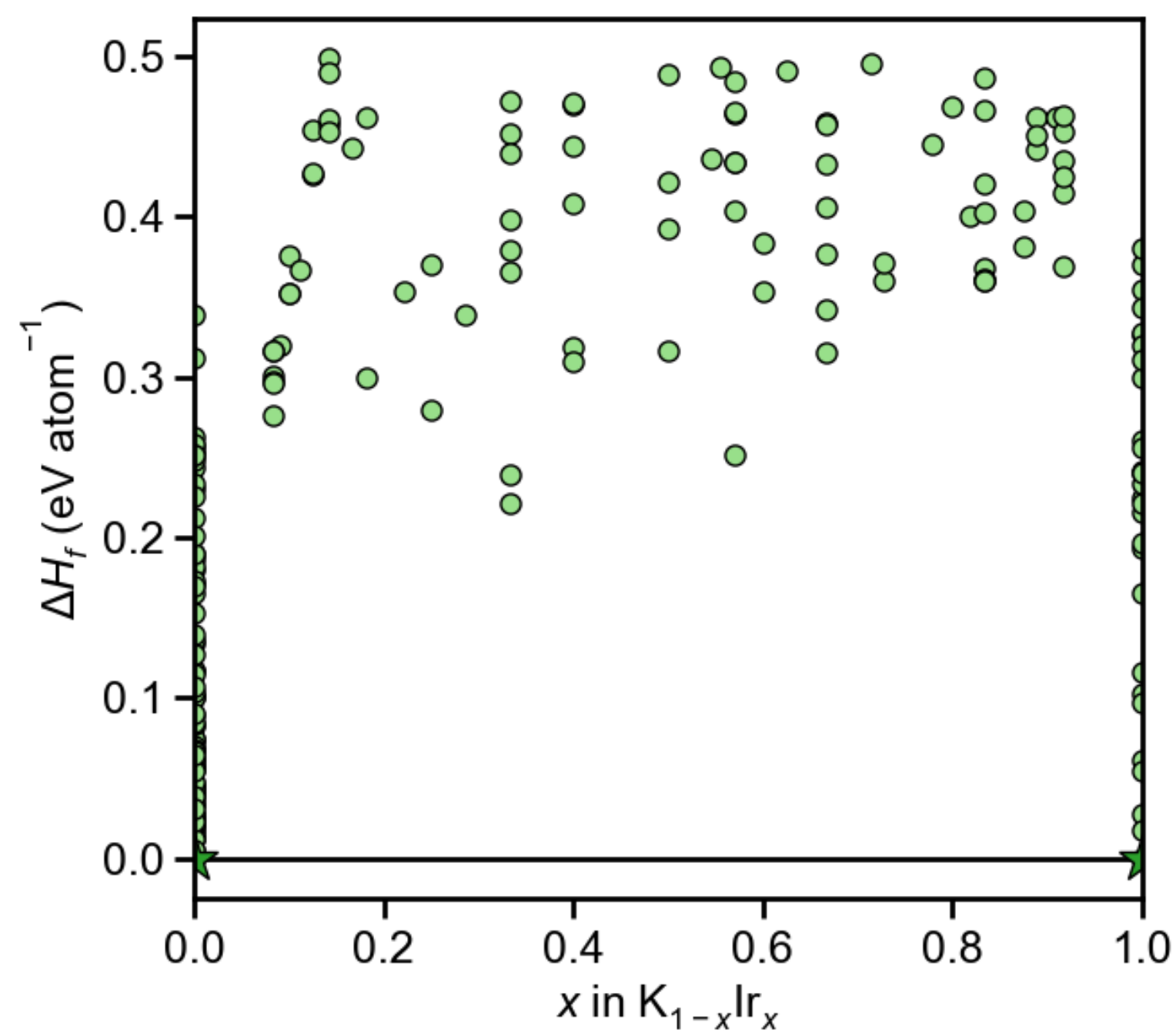

Figure S12 | Formation enthalpies from zero-temperature crystal structure prediction (DFT–PBE) for the K–Ir system at zero pressure. No stable crystalline compounds are predicted.

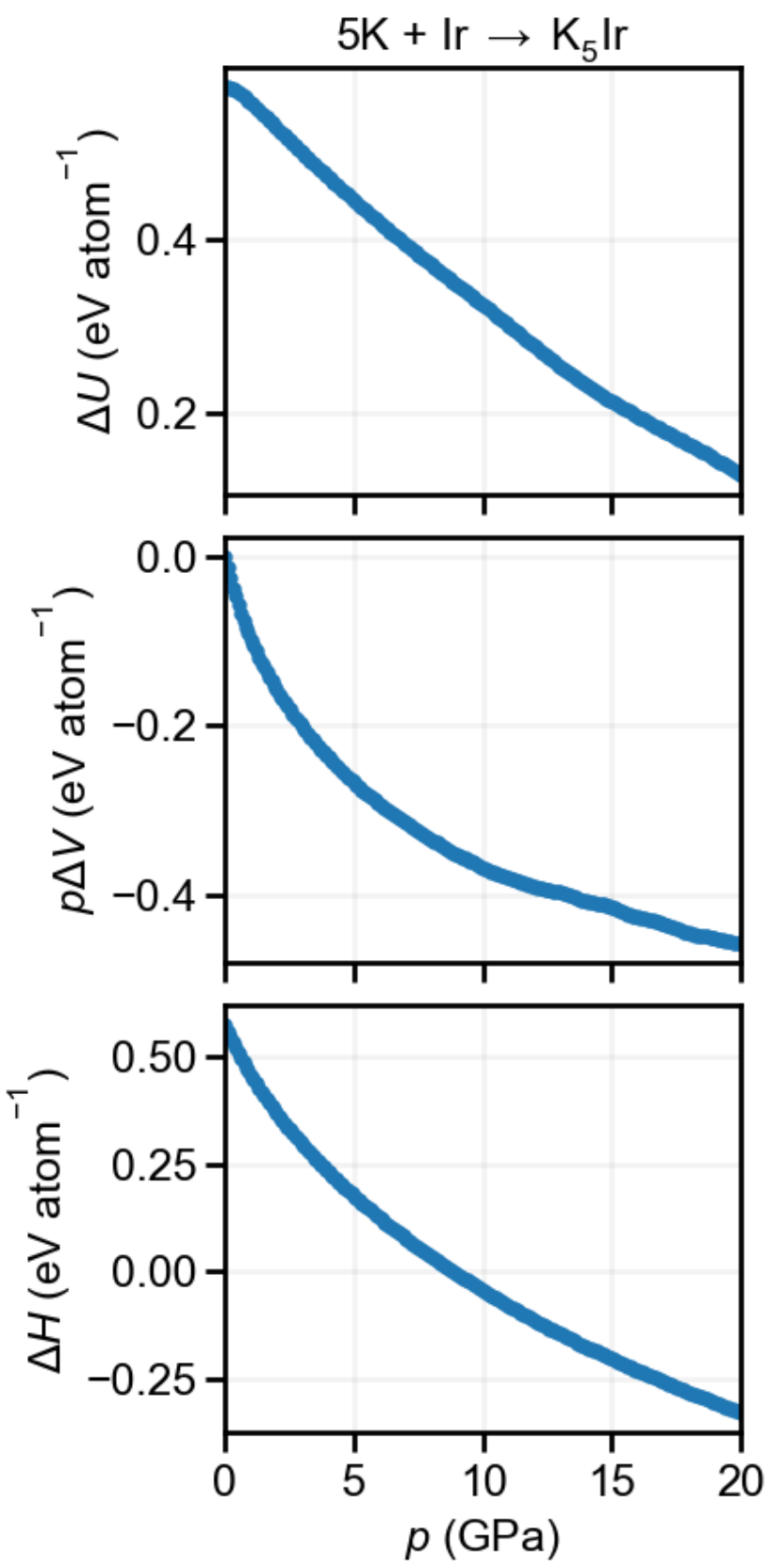

Figure S13 | Computed thermodynamics (DFT–PBE) of K_5Ir formation as a function of pressure. The formation enthalpy is positive below ~8 GPa, indicated thermodynamic instability of K_5Ir against the elements.

References

[1] V. B. Prakapenka, A. Kubo, A. Kuznetsov, A. Laskin, O. Shkurikhin, P. Dera, M. L. Rivers, S. R. Sutton, “Advanced flat top laser heating system for high pressure research at GSECARS: application to the melting behavior of germanium,” *High Pressure Res.* **2008**, *28*, 225–235.

[2] L. Burakovsky, N. Burakovsky, M. J. Cawkwell, D. L. Preston, D. Errandonea and S. I. Simak, *Phys. Rev. B* **2016**, *94*, 094112.

[3] N. Holtgrewe, E. Greenberg, C. Prescher, V. B. Prakapenka, A. F. Goncharov, “Advanced integrated optical spectroscopy system for diamond anvil cell studies at GSECARS,” *High Pressure Res.* **2019**, *39*, 457–470.

[4] Y. Akahama, H. Kawamura, “Pressure calibration of diamond anvil Raman gauge to 310 GPa,” *J. Appl. Phys.* **2006**, *100*, 043516.

[5] C. Prescher, V. B. Prakapenka, “DIOPTAS: a program for reduction of two-dimensional X-ray diffraction data and data exploration,” *High Pressure Res.* **2015**, *35*, 223–230.

[6] B. H. Toby, R. B. Von Dreele, “GSAS-II: the genesis of a modern open-source all purpose crystallography software package,” *J. Appl. Crystallogr.* **2013**, *46*, 544–549.

[7] A. D. Rosa, G. Garbarino, J. E. Rodrigues, E. Mijit, J. Jacobs, D. Bugnazet, S. Pasternak, G. Berruyer, A. Moyne, C. Clavel, F. Perrin, S. Anzellini, C. Meneghini, F. Occelli, X. Zhan, N. Ishimatsu, T. Sakai, S. Boccato, R. Torchio, J.-A. Hernandez, C. J. S. Heath, S. Dominijanni, G. Morard, D. Antonangeli, S. Petitdemange, B. Wehinger, M. Mezouar, G. Kovalskii, W. Morgenroth, M. Wilke, A. Di Cicco, M. A. Bouhifd, T. Irifune, K. A. Lomachenko, O. Mathon, “New opportunities for high pressure X-ray absorption spectroscopy at ID24-DCM and BM23 with the Extremely Brilliant Source of the ESRF,” *High Press. Res.* **2024**, *44*, 248–276.

[8] G. Kresse, J. Hafner, “Ab initio molecular dynamics for liquid metals,” *Phys. Rev. B* **1993**, *47*, 558–561.

[9] G. Kresse, J. Hafner, “Ab initio molecular-dynamics simulation of the liquid-metal–amorphous-semiconductor transition in germanium,” *Phys. Rev. B* **1994**, *49*, 14251–14269.

[10] G. Kresse, J. Furthmüller, “Efficiency of ab-initio total energy calculations for metals and semiconductors using a plane-wave basis set,” *Comput. Mater. Sci.* **1996**, *6*, 15–50.

[11] G. Kresse, J. Furthmüller, “Efficient iterative schemes for ab initio total-energy calculations using a plane-wave basis set,” *Phys. Rev. B* **1996**, *54*, 11169–11186.

[12] P. E. Blöchl, “Projector augmented-wave method,” *Phys. Rev. B* **1994**, *50*, 17953–17979.

[13] G. Kresse, D. Joubert, “From ultrasoft pseudopotentials to the projector augmented-wave method,” *Phys. Rev. B* **1999**, *59*, 1758–1775.

[14] J. P. Perdew, K. Burke, M. Ernzerhof, “Generalized Gradient Approximation Made Simple,” *Phys. Rev. Lett.* **1996**, *77*, 3865–3868.

[15] J. Heyd, G. E. Scuseria, M. Ernzerhof, “Hybrid functionals based on a screened Coulomb potential,” *J. Chem. Phys.* **2003**, *118*, 8207–8215.

[16] T. M. Henderson, J. Paier, G. E. Scuseria, “Accurate treatment of solids with the HSE screened hybrid,” *Phys. Status Solidi B* **2010**, *248*, 767–774.

[17] D. Hobbs, G. Kresse, J. Hafner, “Fully unconstrained noncollinear magnetism within the projector augmented-wave method,” *Phys. Rev. B* **2000**, *62*, 11556–11570.

[18] S. Steiner, S. Khmelevskyi, M. Marsmann, G. Kresse, "Calculation of the magnetic anisotropy with projected-augmented-wave methodology and the case study of disordered $Fe_{1-x}Co_x$ alloys," *Phys. Rev. B* **2016**, *93*, 224425.

[19] N. Marzari, D. Vanderbilt, "Maximally localized generalized Wannier functions for composite energy bands," *Phys. Rev. B* **1997**, *56*, 12847–12865.

[20] I. Souza, N. Marzari, D. Vanderbilt, "Maximally localized Wannier functions for entangled energy bands," *Phys. Rev. B* **2001**, *65*, 035109.

[21] G. Pizzi, V. Vitale, R. Arita, S. Blügel, F. Freimuth, G. Géranton, M. Gibertini, D. Gresch, C. Johnson, T. Koretsune, J. Ibañez-Azpiroz, H. Lee, J.-M. Lihm, D. Marchand, A. Marrazzo, Y. Mokrousov, J. I. Mustafa, Y. Nohara, Y. Nomura, L. Paulatto, S. Poncé, T. Ponweiser, J. Qiao, F. Thöle, S. S. Tsirkin, M. Wierzbowska, N. Marzari, D. Vanderbilt, I. Souza, A. A. Mostofi, J. R. Yates, "Wannier90 as a community code: new features and applications," *J. Phys. Condens. Matter* **2020**, *32*, 165902.

[22] A. G. Eaton, T. I. Weinberger, N. J. M. Popiel, Z. Wu, A. J. Hickey, A. Cabala, J. Pospíšil, J. Prokleška, T. Haidamak, G. Bastien, P. Opletal, H. Sakai, Y. Haga, R. Nowell, S. M. Benjamin, V. Sechovský, G. G. Lonzarich, F. M. Grosche, M. Vališka, "Quasi-2D Fermi surface in the anomalous superconductor UTe_2," *Nat. Commun.* **2024**, *15*.

[23] W. Davey, F. Wick, "Crystal structures of CsCl and TlCl," *Phys. Rev* **1921**, *17*, 403.

[24] W. E. Spicer, A. H. Sommer, J. G. White, "Studies of the Semiconducting Properties of the Compound CsAu," *Phys. Rev.* **1959**, *115*, 57–62.

[25] G. Gnutzmann, F. Wilhelm Dorn, W. Klemm, "Das Verhalten der Alkalimetalle zu Halbmetallen. VII. Über einige A_3B- und AB_2-Verbindungen der schweren Alkalimetalle mit Elementen der V. Gruppe," *Z. Anorg. Allg. Chem.* **1961**, *309*, 210–225.

[26] L. Westin, L.-E. Edshammar, "The Crystal Structure of Ir_7Mg_{44}," *Acta Chem. Scand.* **1972**, *26*, 3619–3626.

[27] H. Donkersloot, J. Van Vucht, "The crystal structure of IrLi, Ir_3Li and $LiRh_3$," *J. Less Common Met.* **1976**, *50*, 279–282.

[28] H. Sommer, R. Hoppe, "Die Kristallstruktur von Cs_2S mit einer Bemerkung über Cs_2Se, Cs_2Te, Rb_2Se und Rb_2Te," *Z. Anorg. Allg. Chem.* **1977**, *429*, 118–130.

[29] J. E. Ellis, R. A. Faltynek, "The tetracarbonyl trianions of manganese and rhenium, $M(CO)_4^{3-}$," *J. Chem. Soc., Chem. Commun.* **1975**, 966.

[30] K.-J. Range, P. Hafner, "Structure refinement of $AuMg_3$, $IrMg_3$ and $IrMg_{2.8}$," *J. Alloys Compd.* **1993**, *191*, L5–L7.

[31] F. Bonhomme, K. Yvon, "Cubic $Mg_{29}Ir_4$ crystallizing with an ordered variant of the Mg_6Pd-type structure," *J. Alloys Compd.* **1995**, *227*, L1–L3.

[32] J. M. Allen, W. W. Brennessel, C. E. Buss, J. E. Ellis, M. E. Minyaev, M. Pink, G. F. Warnock, M. L. Winzenburg, V. G. Young, "Synthesis, Isolation, and Characterization of Trisodium Tricarbonyliridate (3−), $Na_3[Ir(CO)_3]$. Initial Studies on Its Derivative Chemistry and Structural Characterizations of *trans*-$[Ir(CO)_3(EPh_3)_2]^-$, E = Ge, Sn, and *trans*-$[Co(CO)_3(SnPh_3)_2]^-$," *Inorg. Chem.* **2001**, *40*, 5279–5284.

[33] A. Karpov, J. Nuss, U. Wedig, M. Jansen, "Cs_2Pt: A Platinide(-II) Exhibiting Complete Charge Separation," *Angew. Chem. Int. Ed.* **2003**, *42*, 4818–4821.

[34] V. Hlukhyy, U. C. Rodewald, R.-D. Hoffmann, R. Pöttgen, "Synthesis and Structure of $RhMg_3$ and Ir_3Mg_{13}," *Z. Naturforsch. B* **2004**, *59*, 251–255.

[35] M. Pani, A. Palenzona, "Ca_5Rh and Ca_5Ir: Isotypic compounds with a new structure type," *J. Alloys Compd.* **2007**, *436*, 91–94.

[36] J. Salvador, J. Herbst, M. Meyer, "Synthesis of Li_2MgIr and $LiMgIrH_6$: Guidance from DFT," *J. Alloys Compd.* **2011**, *509*, 3203–3207.

[37] J. Brgoch, M. Hermus, "Pressure-Stabilized Ir^{3-} in a Superconducting Potassium Iridide," *J. Phys. Chem. C* **2016**, *120*, 20033–20039.

[38] T. A. Manz, N. G. Limas, "Introducing DDEC6 atomic population analysis: part 1. Charge partitioning theory and methodology," *RSC Adv.* **2016**, *6*, 47771–47801.

[39] N. G. Limas, T. A. Manz, "Introducing DDEC6 atomic population analysis: part 2. Computed results for a wide range of periodic and nonperiodic materials," *RSC Adv.* **2016**, *6*, 45727–45747.

[40] G. Henkelman, A. Arnaldsson, H. Jónsson, "A fast and robust algorithm for Bader decomposition of charge density," *Comput. Mater. Sci.* **2006**, *36*, 354–360.

[41] E. Sanville, S. D. Kenny, R. Smith, G. Henkelman, "Improved grid-based algorithm for Bader charge allocation," *J. Comput. Chem.* **2007**, *28*, 899–908.

[42] W. Tang, E. Sanville, G. Henkelman, "A grid-based Bader analysis algorithm without lattice bias," *J. Phys. Condens. Matter* **2009**, *21*, 084204.

[43] M. Yu, D. R. Trinkle, "Accurate and efficient algorithm for Bader charge integration," *J. Chem. Phys.* **2011**, *134*.

[44] C. Ertural, S. Steinberg, R. Dronskowski, "Development of a robust tool to extract Mulliken and Löwdin charges from plane waves and its application to solid-state materials," *RSC Adv.* **2019**, *9*, 29821–29830.

[45] R. Nelson, C. Ertural, J. George, V. L. Deringer, G. Hautier, R. Dronskowski, "LOBSTER: Local orbital projections, atomic charges, and chemical-bonding analysis from projector-augmented-wave-based density-functional theory," *J. Comput. Chem.* **2020**, *41*, 1931–1940.

[46] A. D. Becke, K. E. Edgecombe, "A simple measure of electron localization in atomic and molecular systems," *J. Chem. Phys.* **1990**, *92*, 5397–5403.

[47] A. Savin, O. Jepsen, J. Flad, O. K. Andersen, H. Preuss, H. G. von Schnering, "Electron Localization in Solid-State Structures of the Elements: the Diamond Structure," *Angew. Chem. Int. Ed.* **1992**, *31*, 187–188.

[48] B. Silvi, A. Savin, "Classification of chemical bonds based on topological analysis of electron localization functions," *Nature* **1994**, *371*, 683–686.

[49] A. Togo, "First-principles Phonon Calculations with phonopy and phono3py," *J. Phys. Soc. Jpn.* **2023**, *92*, 012001.

[50] A. Togo, L. Chaput, T. Tadano, I. Tanaka, "Implementation strategies in phonopy and phono3py," *J. Phys. Condens. Matter* **2023**, *35*, 353001.

[51] F. Mouhat, F.-X. Coudert, "Necessary and sufficient elastic stability conditions in various crystal systems," *Phys. Rev. B* **2014**, *90*.

[52] A. R. Oganov, C. W. Glass, "Crystal structure prediction using ab initio evolutionary techniques: Principles and applications," *J. Chem. Phys.* **2006**, *124*.

[53] A. R. Oganov, A. O. Lyakhov, M. Valle, “How Evolutionary Crystal Structure Prediction Works—and Why,” *Acc. Chem. Res.* **2011**, *44*, 227–237.

[54] A. O. Lyakhov, A. R. Oganov, H. T. Stokes, Q. Zhu, “New developments in evolutionary structure prediction algorithm USPEX,” *Comput. Phys. Commun.* **2013**, *184*, 1172–1182.

[55] K. Momma, F. Izumi, “VESTA 3 for three-dimensional visualization of crystal, volumetric and morphology data,” *J. Appl. Crystallogr.* **2011**, *44*, 1272–1276.

[56] S. P. Ong, W. D. Richards, A. Jain, G. Hautier, M. Kocher, S. Cholia, D. Gunter, V. L. Chevrier, K. A. Persson, G. Ceder, “Python Materials Genomics (pymatgen): A robust, open-source python library for materials analysis,” *Comput. Mater. Sci.* **2013**, *68*, 314–319.

[57] A. Togo, K. Shinohara, I. Tanaka, “Spglib: a software library for crystal symmetry search,” *Sci. Technol. Adv. Mater. Meth.* **2024**, *4*.

[58] E. Kroumova, M. Aroyo, J. Perez-Mato, A. Kirov, C. Capillas, S. Ivantchev, H.Wondratschek, “Bilbao Crystallographic Server: Useful Databases and Tools for Phase-Transition Studies,” *Phase Transitions* **2003**, *76*, 155–170.

[59] M. I. McMahon, R. J. Nelmes, U. Schwarz, K. Syassen, “Composite incommensurate K-III and a commensurate form: Study of a high-pressure phase of potassium,” *Phys. Rev. B* **2006**, *74*.

[60] L.-g. Liu, “Compression and polymorphism of potassium to 400 kbar,” *J. Phys. Chem. Solids* **1986**, *47*, 1067–1072.

[61] K. Takemura, K. Syassen, “High-pressure phase transitions in potassium and phase relations among heavy alkali metals,” *Phys. Rev. B* **1983**, *28*, 1193–1196.

[62] M. Alouani, N. E. Christensen, K. Syassen, “Calculated ground-state and optical properties of potassium under pressure,” *Phys. Rev. B* **1989**, *39*, 8096–8106.

[63] M. Whangbo, C. Lee, J. Köhler, “Transition-Metal Anions in Solids and Their Implications on Bonding,” *Angew. Chem. Int. Ed.* **2006**, *45*, 7465–7469.

[64] J. E. Ellis, C. P. Parnell, G. P. Hagen, “Highly reduced organometallics. 3. Tetrasodium tetracarbonylmetalates(4-) of chromium, molybdenum, and tungsten, $Na_4M(CO)_4$,” *J. Am. Chem. Soc.* **1978**, *100*, 3605–3607.
